# Racism and Indigenous Adolescent Development: A Scoping Review

**DOI:** 10.1111/jora.12754

**Published:** 2022-04-03

**Authors:** Bep Uink, Rebecca Bennett, Jonathan Bullen, Ashleigh Lin, Gregory Martin, Jenna Woods, Yin Paradies

**Affiliations:** ^1^ 5673 Murdoch University; ^2^ Telethon Kids Institute Perth; ^3^ 1649 Curtin University; ^4^ 1994 University of Technology Sydney; ^5^ 2104 Deakin University

## Abstract

Previous studies on the impacts of racism on adolescent development have largely overlooked Indigenous youth. We conducted a scoping review of the empirical literature on racism against Indigenous adolescents to determine the nature and scope of this research and to establish associations with developmental outcomes. Our literature search resulted in 32 studies with samples from the United States, Canada, Australia and New Zealand. Studies were limited to self‐reported experiences of racism and thus primarily focused on perceived discrimination. Quantitative studies found small to moderate effects of perceived discrimination on adolescent psychopathology and academic outcomes. Qualitative studies provided insight into structural forms of racism. We offer recommendations for future investigations into the impacts of overt and covert racism on Indigenous adolescents.

Negative impacts of racism on adolescent outcomes, including identity development and psychopathology, are well documented in the developmental literature (Benner et al., 2018; Cave et al., 2020a). However, to date, most studies have focused on Black, Latinx/Hispanic, or Asian American (i.e., Black and People of Colour; BPOC) youth, to the exclusion of Indigenous populations. This exclusion limits knowledge about the specific forms of racism experienced by Indigenous peoples and potentially contributes to politically motivated erasures of Indigenous specificities (Smith, 2012). Additionally, existing research appears disproportionately focused on overt forms of racism, which are easily identifiable and deliberately harmful, without a parallel understanding of the impacts of *covert* forms of racism, which can be ambiguous and/or unintentional to perpetrators but have significant impacts on targets (Jones et al., 2016; Sue et al., 2007). If research into systems of racial oppression affecting Black, Indigenous, and People of Colour (BIPOC) adolescents is to advance an increased focus on Indigenous populations, this work must address covert as well as overt racist forms. To support this call, we present a scoping review of the current empirical literature on the relationship between racism and Indigenous youth development in settler‐colonial nations (i.e., Canada, the United States, Australia, and New Zealand). Settler‐colonial nations are defined as areas that experience continuous and ongoing direct colonization as processes and structures (rather than a singular event) which aim to eliminate those peoples who predate colonization (Murrey, 2020). In conducting this review we identify salient gaps in the literature and offer directions to guide future empirical work.

## Theoretical Framework

The developmental theory has long posited that social systems, such as racism, influence adolescent development (e.g., Coll et al., 1996). In their seminal model, Coll and colleagues asserted that racism creates environments disruptive to minority youth development: race acts as a “social positioning factor” that leads to social, economic, and psychological segregation based on false perceptions of (non‐White) racial inferiority. Further, racism creates and maintains “inhibiting” and “supportive” environments that directly impact youth development. For example, racism can be linked‐to comparatively higher proportions of BIPOC adolescents living in environments with reduced economic resources, which can foster maladaptive behaviors such as crime and substance abuse (e.g., Spano et al., 2008). At the same time, supportive environments emerge within these contexts through adaptive cultural mechanisms which buffer the negative influences of racism on development (e.g., ethnic‐racial socialization; Neblett et al., 2012). More recently, developmental scholars have leveraged Intersectionality Theory (Crenshaw, 1990) to highlight how multiple systems of oppression (e.g., racism, ableism, and homophobia) intersect to impede development (Santos & Toomey, 2018).

## Racism Against Indigenous Peoples

Although the aforementioned theories provide a foothold from which developmental science can investigate the impacts of racism on adolescent development they have not been developed with Indigenous youth specifically in mind. Indigenous conceptualizations of racism help articulate unique threats to Indigenous populations, tied to historical and ongoing mechanisms of oppression in settler‐colonial nation states (Brayboy, 2005; Paradies, 2016). Approaches such as Tribal Critical Race Theory (TribCrit; Brayboy, 2005) situate Indigenous peoples' experiences of racism as being nested within settler‐colonialism and imperialism. TribCrit also positions Indigenous peoples in settler‐colonial countries as racialized and “legal/political” beings (p.432) whose contemporary experiences are uniquely shaped by perpetual delegitimization or erasure of Indigenous Knowledge and the ongoing fight for sovereignty.

Positioning racism within settler‐colonialism acknowledges specific forms of racism linked to the measures used by the British Empire in its historical acquisition and ongoing occupation of Indigenous sovereign territories (Short, 2016). One such measure leveraged skin color as a distinctive trope, whereby darker skinned “natives” were positioned as being savage, deviant, or inferior and lighter‐skinned “colonizers” as rational, civil, and superior. This rationale underpins the dominance of race as a social positioning factor, with implications for all BIPOC youth (as per Coll et al., 1996). For Indigenous people specifically, settler‐colonialism creates a shared historical context that connects diverse Indigenous Nations. Different from BPOC, the experience of First Nations peoples across the globe are informed by historical trauma and loss from settler policies of genocide, assimilation, and the forced removal of children. This is exacerbated in the subsequent omission or downplaying, of the brutality of these events in contemporary narratives of settler‐colonial nationhood (Short, 2016).

Findings from racism research with Indigenous populations across Canada, the United States, Australia, and New Zealand are strikingly similar, including low academic expectations of Indigenous students, the invisibility of Indigenous perspectives in school contexts, primitive stereotyping, and questioning peoples' Indigeneity in broader social contexts (Alansari et al., 2020; Clark et al., 2014; Masta, 2018; Moodie et al., 2019). This similarity suggests that, across the globe, Indigenous stereotypes are situated in similar colonial discourse (Brayboy, 2005), and not in the reality of vast diversity in cultural practices and experiences among (and within) Indigenous nations. This discursive denial of heterogeneity supports assumptions that Indigenous experiences of racism can be fully understood through non‐Indigenous racial‐ethnic groups' experiences and may implicitly contribute to the dearth of empirical research focused on Indigenous young people's specific experience of racism.

## The Many Faces of Racism

Racism can be conceptualized in terms of type (i.e., overt vs. covert) and proximity to target (proximal vs. distal). Overt racism is largely condemned in settler‐colonial nations; it has clear intent to disempower, exclude or harm and is thus easy to identify and sanction. Covert racism is harder to track and often manifests as ambiguity and paradox. For example, covert racism is found in both intolerance *and* “tolerance” of racial others (Chong, 1994); in negative *and* “positive” racial stereotypes; in the denial of White privilege; and in well‐meaning deficit discourses about Indigenous populations (Seet & Paradies, 2018). Such covert forms also appear within racial microaggressions (Sue et al., 2007). Although previous research demonstrates the high frequency of covert racism among Indigenous adult populations (e.g., Clark et al., 2014; Masta, 2018), there is limited understanding of the impacts of covert racism on Indigenous adolescents.

## Why Focus on Adolescents?

Studies from settler‐colonial nations indicate that Indigenous adolescents experience poorer mental health outcomes compared to non‐Indigenous adolescents (e.g., Australia: Azzopardi et al., 2018; New Zealand: Crengle et al., 2012; US: Harris et al., 2006). Adolescence offers specific vulnerabilities as well as affordances for responding to racism. During this period, self regulation systems undergo rapid reorganization increasing youths' vulnerability to environmental stressors (Crone & Dahl, 2012; Luciana, 2013). Given that racism is associated with heightened allostatic load among Indigenous peoples (Cave et al., 2020b), race‐based stress may further tax Indigenous adolescents' already overburdened selfregulation systems. The adolescent brain is also acutely focused on social evaluation during identity development (Somerville, 2013). Hearing negative views about oneself or cultural group via racist acts may therefore have detrimental long‐term impacts on identity and selfconcept (Cave et al., 2020a). Racism can additionally affect adolescent development through maladaptive coping mechanisms (e.g., substance abuse; Davis et al., 2019) which in turn can lead to ostracism from community and family networks, disrupting central tenants of Indigenous well‐being (Gee et al., 2014). Despite specific vulnerabilities, adolescents can have adaptive responses to racism. For example, youth activism can provide important learning opportunities including helping youth develop “critical consciousness” of their cultural or racial group (Akiva et al., 2017; Carey et al., 2020). Participation in culture‐based activism also generates sources of well‐being including connection to ancestral land, the natural environment, and community (Freeman, 2019). Such participation in ongoing cultural practices can buffer the negative consequences of racial discrimination on youth (Currie et al., 2019).

## Previous Reviews

Previous literature reviews focused on the developmental impacts of racism among adolescents have linked racism with a plethora of health outcomes, including psychopathology and low selfesteem (Benner et al., 2018; Cave et al., 2020a; Priest et al., 2013). Although they included Indigenous samples, these reviews did not elucidate specific or comparative impacts for these groups, nor did they acknowledge the specific Indigenous Nations represented within each sample. The small number of reviews with exclusively Indigenous youth populations (Alansari et al., 2020; Moodie et al., 2019) have specifically considered racism within school contexts, but not within other salient developmental contexts. In addition, previous reviews have not had an explicit reference to culturally grounded methodology, which is considered vital for understanding Indigenous interpretations of health and development (Walters et al., 2020). Culturally grounded methodologies are those framed by Indigenous worldviews, both in relation to scientific inquiry and the phenomenon under investigation, and require that study formulation, design, procedures, and data analysis are situated within Indigenous worldviews. Culturally grounded investigations include leadership or collaboration with Indigenous peoples from study inception to dissemination and implementation of findings. To address these gaps, this scoping review aimed to examine the empirical literature focused exclusively on Indigenous adolescent populations in settler‐colonial nations across all social and institutional contexts, making explicit note of culturally grounded methodologies when applied.

## Search Strategy

After consultation with a subject expert librarian, we limited the search to databases deemed the most likely to include developmental studies involving Indigenous adolescents (see Table S1 for details of piloting the search strategy). The reference lists of six published reviews on racism, microaggressions, and health were also manually scanned for studies that met the inclusion criteria. To develop a comprehensive understanding of the research landscape, no date limits or developmental outcomes were specified in the search string. Both qualitative and quantitative studies, addressing all forms of racism, were included for the same reason.

### Inclusion Criteria

The Population, Concept, and Context (PCC) for study inclusion were adolescents aged 12–25 years (see note for Table S1); studies had to focus on the concepts of *racism* (including racial discrimination, covert and overt racism, interpersonal racism, structural/systematic racism, and microaggressions) and *adolescent development*. We took a broad definition of development so that studies were included if they examined salient tasks of adolescence (i.e., identity development, development of autonomy, peer and romantic relations, and educational attainment) or risks specifically associated with adolescence (i.e., internalizing and externalizing psychopathology). Consistent with previous reviews (Benner et al., 2018; Cave et al., 2020a), only self‐(or other) reports of subjective experiences of racism were included, omitting studies focused on perpetrator behavior or policy analysis. Consistent with past research (e.g., Jongen et al., 2020), the search strategy was limited to youth from four settler‐colonial nation states: the United States, Canada, Aotearoa (New Zealand), and Australia.

## Results

**Figure 1:**
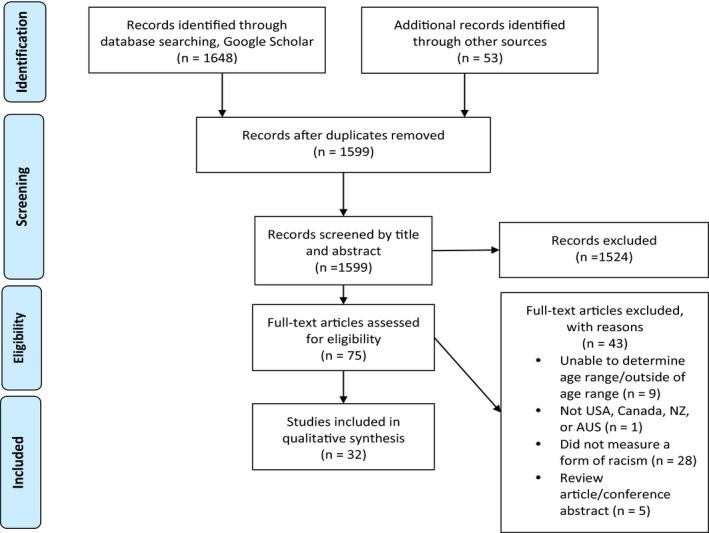
PRISMA flow diagram of included and excluded studies.

Thirty‐two studies met the inclusion criteria. Figure 1 (below) illustrates the total number of records screened after the search strategy had been piloted and finalized. Table S2 provides details of each study. Thirty studies were published in peer‐reviewed journals and two were dissertations (Chen, 2003; Middlebrook, 2010). Below, we review the following components of included studies: (1) participant characteristics, (2) study recruitment, (3) methodologies, (4) how racism was assessed, (5) impacts on development, (6) multiethnic comparisons, and (7) mediators and moderators of relations between racism and developmental outcomes.

### Nature and Characteristics of Indigenous Adolescent Racism Research

#### Participant characteristics

The majority of studies (*n* = 11) included Native American participants (USA), followed closely by Aboriginal and Torres Strait Islander (AUS) youth (*n* = 10). Five studies were exclusive with First Nations Canadian (CAN) youth and an additional five included both the United States and Canadian populations. Only one study included Māori (NZ) youth. Seven studies included participants under the age of 12 (Armenta et al., 2016; Chen, 2003; Greenfield et al., 2017; Melander et al., 2013; Nelson & Hay, 2010; Whitbeck et al., 2001, 2002). Chen (2003) and both studies by Whitbeck et al. reported relatively equal proportions of participants across grades five through eight. We were unable to determine the proportion of participants below 12 years in Melander et al. (2013). The remaining studies with participants <12 years followed these participants longitudinally and thus participants were all within the adolescent age range upon completion of the study. Three studies (Galliher et al., 2011; Jaramillo et al., 2016; Middlebrook, 2010) included participants up to 19 years old, two studies (Hare & Pidgeon, 2011; Priest, Paradies, Gunthorpe et al., 2011; Priest, Paradies, Stewart, et al., 2011) included participants up to 20 years old, and one study (Garrett et al., 2017) included participants >19 years (.42% of the sample). The remaining studies (*n* = 17) included youth aged 12–18 years.

Only five studies specified the tribal/language groups included in the Indigenous sample: namely, Inuit, Metis, Puebelo, Anishinaabe, Navajo, and Wikwemikong peoples were acknowledged. Thus, the majority of studies leveraged pan‐Indigenous nomenclature relevant to the region, for example, Native American (USA), First Nations (CAN), Aboriginal and Torres Strait Islander (AUS), and Māori (NZ), without specifying tribes/Nations.

#### Recruitment

Native American and First Nations Canadian youth were largely recruited through research partnerships with local Reservations (see Table S2). In contrast, Aboriginal and Torres Strait Islander and Māori participants were recruited from local high schools, or data were drawn from preexisting datasets. Notably, 13 of the 32 studies appeared to use data from three main data sets: six of the North American studies were based on data that was from the same, large‐scale study of three Native American and four First Nations Canadian Reservations (Armenta et al., 2016; Greenfield et al., 2017; Hartshorn et al., 2012; Martinez & Armenta, 2020; Melander et al., 2013; Walls et al., 2016). These six studies appeared to be based on Whitbek's et al. (2001, 2002) earlier studies among three of these Reservations. The three studies by Bodkin‐andrews et al. (2010a, 2010b, 2013) were based on survey data from five high schools in Eastern Australia which were presumed to be the same high schools. Likewise, Priest et al. (2016, 2019) both drew data from the Longitudinal Study of Australian Children.

#### Methodologies

The majority (75%) of studies employed quantitative survey methodology. Surveys were often administered verbally to maximize comprehension. Eight studies included both youth and caregiver reports (Greenfield et al., 2017; Hartshorn et al., 2012; Hopkins et al., 2014; Martinez & Armenta, 2020; Priest et al., 2016, 2019; Walls et al., 2016; Whitbeck et al., 2002), although there was no caregiver report of youths' experiences of racism. Seven of the remaining studies were qualitative (i.e., utilized a mix of one‐on‐one and focus group interviews [*n* = 6], talking circles [*n* = 1], and observation [*n* = 3]). Johnston‐Goodstar and VeLure Roholt (2017) offered the only mixed‐methods study, which included observations, survey, and analysis of Minnesota Historical Society data.

##### Culturally grounded methodologies

The majority of studies (*n* = 26) reported at least one aspect of culturally grounded methodology (see Table S2). Culturally grounded aspects included: obtaining local Indigenous ethics committees' endorsement (e.g.. Galliher et al., 2011; Priest, Paradies, Gunthorpe et al., 2011; Priest, Paradies, Stewart, et al., 2011), testing culturally specific covariates (e.g., Davis et al., 2019), forming research partnerships with local Reservations (e.g., Whitbeck et al., 2001, 2002), consulting Tribal Advisory Boards (e.g., Chen, 2003), using Indigenous theories (e.g., Harre & Pidgeon, 2011; Quijada Cerecer, 2013), or using research methodologies developed by local Indigenous communities (e.g., Schinke et al., 2010). Quijada Cerecer (2013) framed their research questions and analysis using TribCrit (Brayboy, 2005) and leveraged traditional storytelling methodology to encourage youth participants to generate their own questions for focus group interviews. Hare & Pigeon (Hare & Pidgeon, 2011) utilized Kanein'kehaka (Mohawk) scholar, Alfred (2005)'s concept of “new warrior” to frame their investigation of First Nation Canadian youths' school experiences. Schinke et al. (2010) used Talking Circles to allow a “traditional way of bringing people together with the intent of sharing knowledge, experiences, and feelings” (p. 443) and to “exemplify the collective nature of the intended culture” (p. 443). While culturally grounded methodologies were more often aligned with qualitative approaches, several quantitative studies were also informed and endorsed by local Indigenous communities (e.g., Whitbeck et al., 2001, 2002).

### How was Racism Assessed?

There was little explicit focus on covert racism in the studies, so firm conclusions could not be drawn as to specific experiences and mechanisms of covert forms. Only two studies explicitly measured racial microaggressions: Johnston‐Goodstar and VeLure Roholt (2017) identified several types of microaggressions among Native American adolescents, including discrimination from school staff, racial slurs, harsher punishment (than White students) for misbehavior, feeling invisible in the curriculum, being overly monitored by teachers, assumptions of their inferiority, invalidation of historical trauma, and failure of the school to educate students about issues important to Native American peoples. Similarly, Dickerson et al. (2019) found that 17% of Native American adolescents experienced at least one racial microaggression in the last twelve months.

The vast majority (*n* = 23) of studies measured racism via perceived discrimination. The most popular measures were the Perceived Discrimination Measure (PDM; Whitbeck et al., 2001; *n* = 6 studies), which includes three subscales; *global discrimination*; *authority discrimination*; and *school*, and the Schedule of Racist Events (SRE; Landrine & Klonoff, 1996; *n* = 6 studies) adapted for Indigenous groups. The frequency of perceived discrimination varied across the studies but always fell within the low to mid ranges (see Table S2 for descriptives). Frequencies of racist experiences more broadly varied across studies, ranging from 18% (Arim et al., 2020) to 50% of participants (Chen, 2003) experiencing a racist act in the last year. Notably, a large proportion of Native American participants in both Garrett et al. (2017) and Chen (2003) reported never experiencing racial discrimination, and the majority of those who did in Garrett et al., found it to be “not very disturbing.” In both the SRE and the PDM overt and covert racist events were aggregated to establish a final score, so the differential effects of covert versus overt racism on developmental outcomes could not be isolated. However, descriptive data from the PDM from Chen (2003) and Whitbeck et al. (2001) suggested relative equivalence in the frequency of experiences of overt racist acts (e.g., racial slurs) and covert racist acts (e.g., being followed in shops or teacher surprise at academic competency). Structural racism was largely acknowledged in the qualitative studies (Hare & Pidgeon, 2011; MacDonald, 2019; Quijada Cerecer, 2013; Schinke et al., 2010; Zinga & Gordon, 2016) by highlighting the systemic exclusion of Indigenous perspectives and the over‐pathologizing of Indigenous students in school contexts. Only one quantitative study (Bodkin‐Andrews et al., 2010b) included a measure of “Macro‐Discrimination” (e.g., “Other Australians do not care about the hardships faced by people of my culture”). However, like covert racism, this was aggregated with scores on their personal discrimination subscale for predictive analyses. Finally, one study (MacDonald, 2019) included internalized racism, whereby Māori girls reported attributing academic failure and selfdepreciating comments to being Māori.

### Impacts of Racism on Indigenous Adolescent Development

#### Academic outcomes

Fifteen studies examined academic outcomes (e.g., achievement and school disengagement). Eight of these were quantitative and included samples from Australia (*n* = 3), Canada (*n* = 2), and the United States (*n* = 3). Only the Australian studies focused exclusively on school contexts, and all found negative associations between racism and academic outcomes, including achievement, engagement, and academic selfsabotage, with small to moderate effect sizes (see Table S2). There was a relatively even spread in qualitative studies that tested academic outcomes across the regions (USA *n* = 2; AUS *n* = 2; CAN *n* = 2; and NZ *n* = 1). These studies also associated racism with negative impacts in the academic domain. For example, Johnston‐Goodstar and VeLure Roholt (2017) and Quijada Cerecer (2013) found that Native American students experienced teacher discrimination, racial slurs, invisibility in curriculum, over‐monitoring and sanctioning, assumptions of inferiority, and invalidation of historical trauma at school; all of which inhibited their ability to celebrate their cultural identity at school. Similar experiences were reported in Australian, Canadian, and New Zealand schools (Edwards‐Groves, 2008; Hare & Pidgeon, 2011; MacDonald, 2019; Nelson & Hay, 2010; Zinga & Gordon, 2016). In Australia (Nelson & Hay, 2010), Aboriginal and Torres Strait Islander adolescents reported experiencing racial slurs, perceptions that teachers did not trust them with class tasks, and having their Aboriginality questioned because they appeared “too White.” In spite of these experiences, students also expressed optimism about their school careers.

#### Externalizing and internalizing symptoms

Fifteen studies focused on the impacts of racism on externalizing symptoms, internalizing symptoms, or both (NA = 14; AUS = 1), Substance use and Conduct Disorder were the dominant externalizing behaviors measured. Dickerson et al. (2019) found that perceived discrimination was associated with 2.49 times the odds of reporting cigarette use in the past year among Native American adolescents. Armenta et al. (2016) found experiences of racism in early adolescence predicted the development of alcohol use disorder above other important covariates (e.g., peer influence). Similarly, Greenfield et al. (2017) found that perceived discrimination at ages 10–12 was associated with a “high Conduct Disorder‐later‐onset alcohol use disorder” trajectory among adolescents. Hartshorn et al. (2012) findings reiterated that earlier perceived discrimination predicted later externalizing symptoms (aggression) among Native American and First Nations Canadian youth over a 5 years period. Regarding internalizing symptoms, perceived discrimination was found to increase the risk of belonging to “high‐increasing” but also a “high‐decreasing” depression trajectory among Native American adolescents (Martinez & Armenta, 2020), and to be positively associated with suicide risk and poor overall mental health (Priest, Paradies, Gunthorpe et al., 2011; Priest, Paradies, Stewart, et al., 2011).

#### Bullying

Four studies (CAN = 1; NA = 1; and AUS = 2) examined relations between racism and bullying (Arim et al., 2020; Melander et al., 2013; Priest et al., 2019; Priest et al., 2016). Arim et al. (2020) found that First Nations Canadian youth who perceived high levels of bullying at school were likely to report high levels of perceived discrimination; bullying was then linked to psychological distress and lifetime suicidal ideation. Melander et al. (2013) also found that perceived discrimination was associated with being a bully, a victim, and a victim/perpetrator. In Australia, Priest et al. (2016) found low concordance between bullying and perceived discrimination measures, suggesting that they were separate and discrete stressors. In a subsequent study, higher levels of both bullying and discrimination were reported by Indigenous students, compared to other ethnic minorities (Priest et al., 2019).

#### Identity and selfesteem

An additional four studies (Bodkin‐Andrews et al., 2010a, 2013; Galliher et al., 2011; Macdonald, 2019) tested associations between racism and selfesteem, selfconcept, or identity. Surprisingly, Bodkin‐Andrews et al. (2010a, 2013) found perceived discrimination was *not* associated with Aboriginal and Torres Strait Islander adolescents' selfesteem and Galliher et al. (2011) found few longitudinal effects of perceived discrimination on Native American adolescents' selfesteem and social functioning. However, in Macdonald's (2019) qualitative study, Māori participants struggled to connect with their Indigenous identity in schooling embedded in Eurocentric notions of academic success.

#### Resilience and coping

Evidence of adaptive resilience and coping strategies in response to racism was found in three studies across Canada (*n* = 1) and North America (*n* = 2). Schinke et al. (2010) noted that Wikwemikong youth athletes in Canada employed active and passive coping, such as ignoring, avoiding, educating perpetrators, and challenging racism. Hare and Pidgeon (2011) found Anishinaabe Native American youth developed several forms of resistance to interpersonal and structural racism at school, including the adoption of a “warrior stance.” One quantitative study (Davis et al., 2019) in another Native American cohort found less adaptive coping mechanisms such as “drinking to cope” and “drinking for pleasure” were related to higher perceived discrimination.

### Multiethnic comparison

In terms of comparative data sets, Bodkin‐Andrews et al., (2010a, 2010b, 2013) found that Aboriginal and Torres Strait Islander students reported significantly higher rates of personal discrimination, macrodiscrimination, and teacher discrimination, compared to nonIndigenous students (White and ethnic minorities). Further, discrimination was a stronger predictor of school disengagement and academic achievement—and accounted for greater variance in academic outcomes and selfsabotaging behavior—for Indigenous students compared to the other cohorts. In a different Australian study, Sahdra et al. (2020) found that Indigenous youth experienced higher levels of discrimination compared to White adolescents, but similar levels of discrimination as ethnic minorities. Middlebrook (2010) found that Native American adolescents reported higher discrimination and barriers to employment compared to European Americans. Garrett et al. (2017) reported that youth who identified as both Cherokee and White reported higher discrimination than exclusively Cherokee or White identified subsamples.

### Mediation and Moderation Effects

Two studies (Hartshorn et al., 2012; Whitbeck et al., 2001) found evidence of anger as a mediator between perceived discrimination and externalizing symptoms. In a longitudinal study, Hartshorn et al. (2012) demonstrated that anger partially mediated the association between prior perceived discrimination and later aggression among Native American and First Nations Canadian youth. In a multiple‐mediator model, Whitbeck et al. (2001) found that perceived discrimination was associated with anger, which in turn was related to delinquent behaviour and subsequently substance abuse. However, Whitbeck and colleagues’ findings should be interpreted with caution as mediation was not tested longitudinally.

Seven studies included examinations of moderation effects (Armenta et al., 2016; Bodkin‐Andrews et al., 2013; Chen, 2003; Dickerson et al., 2019; Galliher et al., 2011; Garrett et al., 2017; Jaramillo et al., 2016). Armenta et al. (2016) and Garrett et al. (2017) found no evidence for putative moderators (i.e., peer drinking, selfesteem, and ethnicity). Chen (2003) reported counter‐intuitive findings that perceived discrimination was associated with *lower* anger in the context of high peer delinquency, and Dickerson et al. (2019) were unable to test the effects of Traditional Spiritual Activities (TSAs) due to high levels of TSA participation across the sample. Bodkin‐Andrews et al. (2013) found evidence that high levels of multiculturation, as a measure of perceived acceptance of Indigenous cultural identity in school, interacted with teacher discrimination to produce a *stronger* relationship with selfsabotaging behavior. Further, Jaramillo et al. (2016) found lower ethnic identification levels were associated with a stronger relationship between discrimination and hopelessness, suggesting weak ethnic identification may be a risk factor for Indigenous youth. Galliher et al. (2011) tested gender differences in a Native American sample and found that low White American identification ameliorated otherwise negative impacts of discrimination on boys' social functioning and substance use, and girls' selfesteem. In contrast, strong American Indian identification was associated with a positive association between perceived discrimination and social functioning over time for both genders.

## Discussion

We aimed to evaluate the current empirical research examining the impacts of racism on Indigenous adolescent development. Our review was guided by Indigenous‐led arguments that racism against Indigenous peoples is uniquely situated in settler‐colonialism (Brayboy, 2005; Paradies, 2016). Only 32 studies with Indigenous adolescent populations—spread across four national contexts—were returned in response to the search criteria. The majority of these studies focused on explicit, overt forms of racism. Thus, greater empirical focus is needed across all aspects of racism and Indigenous adolescent development—at global, national, local tribe/Nation, and institutional levels—before any tangible conclusions might be drawn. This need provides a caveat for the following analysis, in that even the most dominant areas of attention are underdeveloped and need further study to validate their impact in this area of inquiry. Having said that, analysis of the studies reviewed—in light of theoretical insights offered in TribCrit (Brayboy, 2005), culturally grounded methodology (Walters et al., 2020) and Indigenous models of well‐being (Gee et al., 2014)—finds three key opportunities to grow the evidence base for understanding impacts of racism on Indigenous youth development: (1) measuring the racism spectrum; (2) decolonizing global Indigenous stereotypes; and (3) including Indigenous models of well‐being in developmental outcomes.

### Measuring the Racism Spectrum

Empirical research is yet to adequately capture the complexity and contextuality of racism faced by Indigenous peoples (Benner et al., 2018). In our review, we found that qualitative studies were generally better placed to work with this complexity; however, they are not equipped to determine important measures of frequency and extent of developmental impacts of the racism spectrum. Four studies considered microaggressions, one explicitly referred to covert racism, and three mentioned structural or macroaggressions. However, the majority of studies reviewed defaulted to dominant understandings of racism, which are interpersonal and overt. Further, eight studies from the North American continent were drawn from a single data set with perceived discrimination scales that merged overt and covert forms into a single perceived discrimination score. Leaving covert racism undocumented or absorbed into broader racism measures leaves little understanding of the unique impacts of systemic and interpersonal microaggressions on Indigenous youth development, despite data in this review also suggesting that such forms occur at similar frequencies to overt racism. This limits policy‐makers and those supporting youth in their ability to educate about, and prevent, covert racism.

Overt and covert racism are not mutually exclusive; rather, they are reinforcing constructs where internalized and interpersonal racist acts are nested within a broader macrosystem of structural racism and white supremacy (Seet & Paradies, 2018; Sue et al., 2007). Measures of perceived discrimination, popular among the studies reviewed here, focus on behavioral racism (i.e., treatment), at the exclusion of attitudinal (implicit and explicit personal bias, Clark et al., 1999), subtle (positive stereotyping and exceptionalism; Williams, 2020), and structural racism (institutional, governmental, economic bias; Berman & Paradies, 2010). Perceived discrimination scales rely on participant identification of unfair treatment being racially motivated. As subtle racism is often ambiguous (Williams, 2020), it is possible that discriminatory treatment that is not obviously attributed to racial bias (yet may have significant cumulative impacts on developmental outcomes) is being overlooked. Modern, subtle, and covert forms of racism, such as racial microaggressions, are a growing field of inquiry in terms of their impacts on BIPOC populations (Jones et al., 2016). Some scales specifically targeting microaggressions among adolescents have been developed (e.g., English et al., 2020), but require testing and adaptation with Indigenous samples. To complement perceived discrimination measures, testing and adaptation of a broader range of scales that concurrently assess covert, overt, and systemic racism will be necessary if scholars are to capture the interplay between these forms.

### Decolonizing Global Indigenous Stereotypes

In settler‐colonial societies, many of which claim to have equality in law, Indigenous peoples continue to be subjected to subtle processes of colonization and the delegitimization of their worldviews (Paradies, 2016). This subtle colonizing process was reflected in studies that did not acknowledge specific Indigenous tribes/Nations represented within their “Indigenous” samples, even when culturally grounded methodologies were employed. While one study withheld naming the specific tribe, upon request of Tribal Advisors (Greenfield et al., 2017), other reports that employed vague or homogenizing cultural categories, such as Indigenous, Aboriginal and Torres Strait Islander, Native American, or First Nations, did not necessarily acknowledge the limitations of this for capturing the diversity between and within tribes/Nations. Thus, there is an opportunity for research to counter homogenizing colonizing forces by conducting studies with distinct tribes/nations, or to acknowledge and name tribes/nations represented within broader study samples.

Notably, different national contexts focused on different outcomes, with the United States and Canada focusing on externalizing and internalizing symptoms, and Australia and New Zealand on academic outcomes. The reasons for these differentials in focus are possibly guided by national policy priorities. For example, in Australia, the national “Closing the Gap” campaign is acutely focused on ameliorating educational disadvantage as a priority area for Aboriginal and Torres Strait Islander youth. Further, racism is rarely openly addressed in politics or education with White normativity and color evasiveness characterizing the school curriculum (Yared et al., 2020). This meant that while experiences of perceived discrimination were linked to internalizing and externalizing symptoms, it was difficult to determine important nuances in the experiences of geographically and culturally distinct populations.

That said, the experience of living in settler‐colonialism has many commonalities across Indigenous populations with an overarching commonality being the systemic erasure of Indigenous history, agency, worldviews, standpoints, and sovereignty in discourses of settler‐colonial nationhood (Short, 2016). Of particular salience to this review is the concept of *omissions* which manifest as the absence of positive Indigenous stereotypes, denial of Indigenous diversity, and favoring of settler histories over Indigenous histories (Fryberg & Eason, 2017). Omissions are a powerful tool of the colonial project whereby continual denial of Indigenous realities serves to perpetuate settler‐colonial narratives of nationhood. Although only a handful of studies in the current review examined omission, which occurred in the context of Indigenous perspectives and histories being overlooked in the school curriculum (e.g., Johnston‐Goodstar & VeLure Roholt, 2017; MacDonald, 2019; Quijada Cerecer, 2013), it was noted as a familiar form of discrimination across all populations. These findings suggest that while there is great diversity in the cultural practices and experiences of Indigenous peoples, they experience the same settler‐colonial system of oppression. Future research should work to unmask and dismantle colonial structures that continue to suppress and erase Indigenous subjectivities, while simultaneously elevating Indigenous voices, methodologies, and worldviews. A helpful first step might be to leverage Indigenous theories (e.g., TribCrit. 2005) as well as emerging models of Indigenous developmental science (Fish, 2021; Fish et al., 2020) to ensure methodological reflexivity and explicit address of the political mechanisms that result in the homogenization or erasure of agency within Indigenous samples.

### Including Indigenous Models of Well‐being in Developmental Outcomes

Overall, our review suggested that racism experienced during adolescence was associated with negative outcomes including poor academic achievement and internalizing and externalizing symptoms. Notably absent in the reviewed studies, however, were conceptualizations of developmental outcomes informed by Indigenous models of well‐being or development. Here, we reflect on our own myopia, as both Indigenous and non‐Indigenous scholars within a settler‐colonial research context, and our need to continually decolonize our own conceptualizations of what constitutes “healthy development.” We acknowledge that we did not include Indigenous‐specific domains of well‐being (e.g., connection to spirit/ancestors, connection to Country; Gee et al., 2014) in our search strategy, meaning that we could have missed important literature in this area. However, our overall sense from the scoping process was that studies with Indigenous‐specified outcomes were absent. This absence also serves as a broader critique of the developmental psychology paradigm which has overlooked holistic, integrated, and spiritual aspects of health (Fish, 2021).

Specifying developmental domains purely from (non‐Indigenous) developmental theory risks overlooking the significant areas which contribute to both risk and thriving among Indigenous youth. As developmental science progresses toward antiracist research practice (https://www.s‐r‐a.org/sra‐statement) Indigenous‐developed models of health and well‐being will be essential for informing culturally specific and culturally relevant outcomes. For example, while Dickerson et al. (2019) was unable to test the conditioning effect of youths' participation in TSAs in the relationship between racism and externalizing symptoms, a culturally grounded approach would view participating in TSAs as a developmental *outcome* of interest, because they represent a connection to culture, spirit, and ancestors. Thus, future research would benefit from the examination of how racism undermines Indigenous youths' ability to participate in TSAs.

This review highlighted the need for more longitudinal studies in Indigenous adolescent racism research to track both moderators and mediators of relations between racism and development. Twelve studies in this review employed longitudinal methods pointing to the feasibility of future longitudinal studies with Indigenous youth populations. Strong ethnic identification was the most commonly studied protective factor in the studies reviewed. Galliher et al. (2011) drew on theoretical arguments that strong ethnic identification buffers against broader societal negative perceptions of Indigenous peoples. Likewise, they suggested that adolescents high in ethnic identification are firmly nested within cultural communities that protect from the impacts of racism. However, Galliher et al. found that the buffering effect of ethnic identification decreased across adolescence, suggesting a risk that ethnic identification decreases in potency as a protective factor across development, perhaps “worn down” by negative societal stereotypes. Previous research finds that participation in youth activist programs affirms minority adolescents' sense of self and identity (Akiva et al., 2017). Youth activist programs may therefore represent developmentally salient contexts to bolster Indigenous adolescents' cultural identity, and ultimately, resilience against racism.^1^

Only two studies in this review examined mediators and found preliminary evidence of anger as a mediator in racism‐externalizing relations. Anger was theorized to result from perceived discrimination due to perceptions of unjustness and discrimination preventing youth from achieving their goals. Anger has also been linked to aggressive and delinquent behavior through General Strain Theory (Agnew, 1992). Thus, anger may have a particular role in determining delinquent behaviors within the context of racism. The danger here is that outward displays of anger by Indigenous youth, especially if linked to aggressive or delinquent behaviors, serve to reinforce negative stereotypes of Indigenous people as “warriors” or aggressors (Fryberg & Eason, 2017). Notably, perceived discrimination was associated with *lower* anger in Chen (2003) for youth in high delinquent peer contexts. This finding suggests that links between perceived discrimination and anger may function differently based on youths' social context.

Our review also pointed toward other mediators worth exploring in the future; the internalization of negative stereotypes impedes an adolescents' selfesteem by reinforcing a belief that Indigeneity equates to “less than” others (Fryberg & Eason, 2017) and low selfesteem has consistently been associated with adolescent psychopathology (Lewinsohn et al., 1994). Thus, future research could explore whether reductions in selfesteem mediate relations between racism and psychopathology outcomes. Given recent evidence linking racism to allostatic load (Cave et al., 2020b) future research should also consider heightened physiological responses to racism as mediating relations with psychopathology. Last, borrowing from Indigenous models of well‐being (Gee et al., 2014) research would benefit from examining relations between exposure to racism → (dis)connection to culture → and well‐being. Indeed, denial of Indigeneity has been documented as a coping response to racism (Mellor, 2004) suggesting that some Indigenous adolescents may actively disengage with cultural communities and activities to avoid racism. This strategy then renders youth unable to benefit from the protective effects of strong ethnic identification (Galliher et al., 2011).

### Limitations

The exclusion of BPOC youth in our search string means some developmental studies with Indigenous youth subsamples may have been missed. However, previous reviews have noted an absence of intergroup comparisons and attributed it to the rarity of Indigenous‐specific studies (e.g., Benner et al., 2018). Second, there was not explicit reference to “internalized racism” or newer terms such as “vicarious racism” (e.g., English et al., 2020) in the search strategy and future reviews may benefit from including these terms. Third, our focus on selfreported experiences of racism meant studies were limited to adolescents' perceptions of unfair treatment and perpetrator intentions. Thus, the suggestion that there was an over‐reliance on perceived discrimination measures may also be attributable to our search strategy limits. Fourth, although we included 32 studies, several drew from the same datasets, which limits generalisability of findings, as well as limiting inter and intragroup comparisons. Finally, only one study was conducted from New Zealand, so no specific or comparative claims can be made as to racism and adolescent development in this region.

### Conclusion

This review offered an overview of the nature, scope, and findings of research examining the developmental impacts of racism among Indigenous adolescents. We found preliminary evidence for the negative impacts of racism (primarily, perceived discrimination) on Indigenous adolescents' academic outcomes and internalizing and externalizing symptoms. Greater breadth and depth of focus on outcomes of racism is needed with both local and international studies to enable cross‐cultural commentary on the frequency and severity of the impact of racism. Likewise, only by considering racism against Indigenous adolescents as embedded within settler‐colonialism and as distinct from other racial and ethnic populations will developmental science be able to fully unveil and unpack this deeply complex, nuanced, and the insidious system of oppression.

## Supporting information


**Table S1.** Search Strings used in Literature Search.

**Table S2.** Summary of Studies Reviewed.

## Footnotes

^1^
While we acknowledge the importance of identifying factors which promote resilience against racism in Indigenous youth we propose that the ultimate goal of racism research is to identity, counter, and dismantle this system of oppression.
