# Supplementary material for: Racism and Indigenous Adolescent Development: A Scoping Review
Source: J Res Adolesc. 2022 Apr 3;32(2):487–500. doi: 10.1111/jora.12754 (PMC9320946; doi:10.1111/jora.12754)
Supplement: Supplementary file 1 — Table S1. Search Strings used in Literature Search. [file JORA-32-487-s001.docx]

Supplementary Table 1: Search Strings used in Literature Search.

| **Database** | **Search string** | **Filters** | **Records** |
| --- | --- | --- | --- |
| PsycINFO | noft((Indian OR Indigenous OR Aborig* OR Torres Strait* OR Pacific* OR Pasifika OR Maori) AND (teen* OR youth OR adolesc*) AND (racis* OR microaggr* OR stereotyp* OR discrim* OR racial OR "covert racism" OR "subtle racism") NOT (Black OR African OR Asian OR Mexic* OR Latin* or Hispanic)) | **Record type**:  Chapter, Dissertation, Journal Article  **Language:**  English  **Age group:**  Adolescence (13-17 Yrs), Young Adulthood (18-29 Yrs)  **Population:**  Human | returned 539 records. |
| ERIC | noft((Indigenous OR first nation* OR native* OR Aborig* OR Torres Strait* OR Maori OR Indian OR Pacific OR Pasifika) AND (subtle racism OR covert racism OR raci* OR microagg*) AND (teen* OR youth OR adolesc* OR school OR child* OR education) NOT (Black OR African OR Asian OR Mexic* OR Latin* OR Hispanic)) | **Language:**  English  **Education level:**  Elementary secondary education, Grade 10, Grade 11, Grade 12, Grade 7, Grade 8, Grade 9, High school equivalency programs, High schools, Higher education, Middle schools, Postsecondary education, Secondary education, Two year colleges  **Applied filters:** NOT (Speeches & Presentations AND Conference Papers & Proceedings AND Encyclopedias & Reference Works) | returned 662 records. |
| Informit | (Indigenous OR indian OR Aborig* OR Torres Strait* OR maori* OR pacific* OR Pasifika) AND (disadvantaged OR racial stereotype OR racial prejudice OR racial discrim* OR race-related stress OR race-related trauma OR racis* OR "covert racism" OR "subtle racism" OR microagg*) AND (youth OR teen* OR adolesc*) | **Search:** ANYWARE  **Filters:**  Peer-reviewed  A+Education collection | returned 149 records but many of them duplicates within the database. |
| SCOPUS | TITLE-ABS-KEY ( indigenous OR aborig* OR torres AND strait* OR first AND nation* OR native* ) AND ( teen* OR youth OR adolesc* ) AND ( racis* OR microagg* OR covert AND racism OR subtle AND racism OR "racial discrim*" ) AND NOT ( black OR african OR mexic* OR latin* OR asian OR hispanic ) | **Language:**  English language.  **Record type:**  journal article, book chapter, **Location:** Australia, Canada, NZ and US. | returned 72 records. |
| CINHAL complete database | ( microagg* OR racism OR racist OR discrim* OR bully* ) AND ( youth OR teen* OR adolesc* ) AND ( Aborig* OR Torres strait* OR Maori OR Indian OR Pacific* OR Pasifika OR Hawaii OR first nation* OR Indigenous OR native* ) NOT ( adult OR man OR woman OR men OR women ) NOT ( Black OR African OR Asian OR Mexic* OR Latin* or Hispanic) | **Search:** ANYWHERE.  **Source type:** Academic journals and dissertations  **Language:** English  **Geography:** Australia, New Zealand, Canada and USA  **Age filter:** Adolescent (13-18 years) | returned 58 records. |
| Proquest Dissertations and Thesis Global | ti(( indigenous OR native* OR first AND nation* OR aborig* OR torres AND strait* OR maori OR pacific* ) ( adolesc* ) OR ( youth* ) OR ( teen* ) ( racis* ) OR ( microagg* ) OR ( subtle AND racism ) OR ( covert AND racism ) OR ( racial AND discrim* ) OR ( racial AND bias ) OR ( racial AND stereotype ) AND NOT ( black ) OR ( african ) OR ( asian ) OR ( mexic* ) OR ( latin* ) or (Hispanic)) | **With filters of:**  Adolescents AND adolescents AND (Native American OR Suicide OR Bullying OR Native Americans OR Stress OR Adolescence) NOT (Latino AND African-American)  English | Returned 29 records |
| Google scholar | 3 sets of advanced searches:   1. racism AND youth Aborig* OR Torres OR Strait OR Maori OR Indian OR Pacific OR Pasifika 2. racism AND youth Aborig* OR Torres OR Strait OR Indian OR Maori OR Pacific OR Pasifika AND "Subtle racism " 3. racism AND youth Aborig* OR Torres OR Strait OR Maori OR Pacific OR Indian OR Pasifka AND "microaggression" | First search:  **With all of the words:** racism AND youth  With **at least one of** the words: Aborig* Torres Strait Maori Indian Pacific Pasifika  **Without** the words: Black African Asian Mexic Latin Hispanic  **Search:** ANYWHERE in the article  Second search: **With the words:** racism AND youth  **With the exact phrase:** subtle racism  **With at least one of the words:** Aborig* Torres Strait Maori Indian Pacific Pasifika  **Without the words:** Black African Asian Mexic Latin Hispanic  **Search:** ANYWHERE in the article  Third search:  **With the words:** racism AND youth  **With the exact phrase:** microaggression  **With at least one of the words:** Aborig* Torres Strait Maori Indian Pacific Pasifika  **Without the words:** Black African Asian Mexic Latin Hispanic  **Search:** ANYWHERE in the article | Returned 10,400 hits. Exported first 100 hits into Endnote.  Returned 29 hits.  Returned 10 hits. |

Notes: Piloting the original search strategy returned a large volume of hits focused on racism among BPOC youth. The qualifiers NOT “Black” OR “African” OR “Asian” OR “Mexic*” OR “Latin*” or “Hispanic” were subsequently added to the search string to reduce the number of out-of-scope research papers. Conversely, the search returned nominal research on ‘adolescence’ specifically (traditionally defined as 12-18 years), so the strategy was expanded to include young adults (19-25 years). The widening of the age range was supported by evidence that brain maturation process in adolescence continue into young adulthood (Tamnes et al., 2010). Database searches were limited to peer-reviewed journal articles, book chapters, reports, and dissertations, and all records had to be published in English language. Search conducted between January and February 2021. Utilising the ‘advanced search’ function in Google Scholar returned <30 hits relevant to this study thus the first 100 hits was deemed enough for the small subject pool, rather than the 200-300 hist recommended by Haddaway et al., (2015).

Reference lists of the following reviews were screened for possible titles:

1. Benner, A. D., Wang, Y., Shen, Y., Boyle, A. E., Polk, R., & Cheng, Y. P. (2018). Racial/ethnic discrimination and well-being during adolescence: A meta-analytic review. *American Psychologist*, *73*(7), 855.
2. Cave, L., Cooper, M. N., Zubrick, S. R., & Shepherd, C. C. (2020). Racial discrimination and child and adolescent health in longitudinal studies: a systematic review. *Social science & medicine*, *250*, 112864.
3. Priest, N., Paradies, Y., Trenerry, B., Truong, M., Karlsen, S., & Kelly, Y. (2013). A systematic review of studies examining the relationship between reported racism and health and wellbeing for children and young people. *Social science & medicine*, *95*, 115-127.
4. Moodie, N., Maxwell, J., & Rudolph, S. (2019). The impact of racism on the schooling experiences of Aboriginal and Torres Strait Islander students: A systematic review. *The Australian Educational Researcher*, *46*(2), 273-295.
5. Alansari, M., Hunia, M., & Eyre, J. (2020). A rapid review of racism in schools.
6. Lui, P. P., & Quezada, L. (2019). Associations between microaggression and adjustment outcomes: A meta-analytic and narrative review. *Psychological bulletin*, *145*(1), 45.

**Supplementary Section References**

Haddaway, N. R., Collins, A. M., Coughlin, D., & Kirk, S. (2015). The role of Google

Scholar in evidence reviews and its applicability to grey literature searching. *PloS one, 10*(9), e0138237.

Tamnes, C. K., Østby, Y., Fjell, A. M., Westlye, L. T., Due-Tønnessen, P., &

Walhovd, K. B. (2010). Brain maturation in adolescence and young adulthood: regional age-related changes in cortical thickness and white matter volume and microstructure. *Cerebral Cortex, 20*(3), 534-548.
