# Supplementary material for: Racism and Indigenous Adolescent Development: A Scoping Review
Source: J Res Adolesc. 2022 Apr 3;32(2):487–500. doi: 10.1111/jora.12754 (PMC9320946; doi:10.1111/jora.12754)
Supplement: Supplementary file 2 — Table S2. Summary of Studies Reviewed. [file JORA-32-487-s002.docx]

Supplementary Table 2: Summary of Studies Reviewed.

| **Study** | **Sample** | **Study Aims(s)/Research Questions(s)** | **Qualitative or Quantitative** | **Study Design and Analysis** | **Form of Racism** | **Evidence of Culturally-grounded research** | **Developmental Outcome** | **Key Findings** |
| --- | --- | --- | --- | --- | --- | --- | --- | --- |
| Arim, et al. (2020). | Data were drawn from the 2012 Aboriginal Peoples Survey, a national, postcensal,  and cross-sectional survey representative of First Nations people living off reserve, Métis,  and Inuit in Canada.  N = 739 youth aged 15-18 years (M*age* = 16.2, SE = .05, 53% male). 21% living in rural areas. | 1) What percentage of First Nations high  school youth living off reserve perceive bullying as a problem at school and are there sex  differences in youth’s perceptions of bullying at school?  2) What are some of the  characteristics of schools (as perceived by youth) in which First Nations youth living off- reserve perceive bullying as a problem?  3) Do First Nations youth living off reserve who perceive bullying as a problem at school have poorer academic (e.g., absenteeism),  behavioural (e.g., drug use), and health (e.g., general and mental health) characteristics  compared with those who do not?  4) Are perceptions of bullying as a problem at  school associated with off-reserve First Nations youth’s mental health even after controlling  for the effects of youth sex, age, and household income? | Quantitative | Cross-sectional, self-report.  Data collected through personal interview or computer assisted interviews. | Asked participants about racism via school climate measure (e.g., the extent to which “racism is a problem at school” “This school supports First Nations, Métis, or Inuit culture through teaching and/or activities” (yes/no). | Used Indigenous specific frameworks to determine covariates of bullying which were meaningful to Indigenous youth (e.g., measured ‘community ties’). Community ties not related to bullying. | Externalizing behaviours (school absenteeism, drug use heavy drinking, smoking), Internalizing symptoms (K10, mood and anxiety disorder), suicidal ideation, academic performance, connection to community. | 37% perceived bullying as a problem; a majority of youth reported feeling safe (97%) and happy (93%) at school; 18% perceived racism at school; 30% perceived violence at school; those who were bullied were less likely to feel safe, safe or enjoy school. Those bullied were more likely to perceive racism, violence, and perceive presence of alcohol and drug at school.  Youth who perceived bullying as a problem at school were statistically significantly more likely to report higher risk for psychological distress (OR=1.74) and lifetime suicidal ideation (OR=1.93) after controlling for the effects of youth sex, age, and household income. |
| Armenta et al. (2016) | Indigenous adolescents in the U.S. Northern Midwest and Canada.  Wave 1 N = 674 (M*age* = 11.11, SD = .83, 50.3% female) 77.6% remained at wave 8. | Examine the antecedents of drinking behaviour among Indigenous adolescents by considering PD and positive drinker prototypes as risk factors for the onset of alcohol use and the development of an Alcohol Use Disorder (AUD). | Quantitative | Interview data from an eight-wave longitudinal study (data collected annually) examining culture-specific risk and resilience factors among Indigenous adolescents. | PD assessed using an11-item measure adapted from the Schedule of Racist Events (Landrine & Klonoff, 1996). Example items: “*how often [in the past year] has a store owner, salesclerk, or person working at a place or business treated you in a disrespectful way because you are[a member of your cultural group]?”“**How often [in the past year] has someone threatened to harm you physically because you are [a member of your cultural group]*? (1 (never) and 3 (many times). | Community partnerships in design and measure selection. Used schedule of racist events adapted for Indigenous youth. | Externalizing psychopathology (onset of alcohol use, AUD) | Average scores for PD were similar across age groups: 11-12 years M =1.24 (0.27); 13-14 years M = 1.21 (0.26); 15-16 years M = 1.21 (0.26).  Increase in alcohol use disorders from age 11-12 to 17-18. 90.1% had tried alcohol and 42.1% had met criteria for lifetime alcohol use disorder by age 17-18.  PD significantly increased the odds of development of AUD (OR = 2.61; B = .12), above and beyond the effects of positive drinker prototypes, and associating with peers who drink.  PD did not influence age of onset of drinking.  After statistically controlling for PD, peer drinking, and gender, the effect of positive drinker prototypes on AUD dropped to a nonsignificant level. |
| Bodkin‐Andrews et al. (2010a) | N = 1,212 high school students (M*age* = 13.63 years, 50.30% male and 49.70% female). 22.50% of the sample were Aboriginal and Torres Strait Islander, 77.48% were non-Indigenous Australians. | 1) To what extent is the factor structure of the measures of PD, general self-esteem, and standardised achievement invariant for Indigenous and non-Indigenous students?  2) How do the latent measures of PD, self-esteem, and the latent interaction between PD and self-esteem independently predict standardised achievement for Indigenous and non- Indigenous students?  3) To what extent are the predictive paths emanating from self-esteem, PD, and the latent interaction factor into maths and spelling achievement equivalent across the Indigenous and non-Indigenous samples? | Quantitative | Cross-sectional survey administered at school. Survey read aloud because of varying reading levels. | The Personal Discrimination Measure (Bodkin-Andrews et al., 2006). A five-item measure designed to assess individuals’ experiences of racial discrimination at the personal (direct contact) level (e.g., ‘*People have called me nasty names based on the culture I come from’*). All items were measured on a 6-point Likert scale, with higher scores indicating greater levels of agreement to experiencing personal discrimination. | N/A | Academic achievement; self-esteem | Indigenous students had higher mean Personal Discrimination score (M = 2.64, SD = 1.32) than non-Indigenous students (M = 1.89, SD = 1.02), although both averages were low, suggesting that for the most part, students disagreed with being discriminated against.  General self-esteem held a negative correlation with Personal Discrimination (although this was only significant for the non-Indigenous students). The weakness of this relation for Indigenous students adds little weight to supporting previous research that has asserted either positive or negative relations between self-esteem and discrimination.    Personal Discrimination negatively predicted spelling achievement and math achievement in both Indigenous and non-Indigenous participants. However, Personal Discrimination explained more variance in Indigenous participants’ academic outcomes (spelling: β= –.23, p< .05, explaining 5.57% of the variance; math: β=–.27, p< .05, explaining 8.10% of the variance) compared to non-Indigenous participants (spelling: β= –.17, p< .05, explaining 3.11% of the variance; maths: β= –.24, p< .05, explaining 6.33% of the variance).  Self Esteem x Personal Discrimination interactions were non-significant. |
| Bodkin‐Andrews et al.(2010b) | N = 278 Indigenous high school students (M*age* =  13.23 years, 57% female,  ).  N = 922 non-Indigenous high school students (M*age* =13.49 years  47% female).  Schools selected as having a minimum of 10% Indigenous enrolment. from five  secondary public schools across four rural and one urban localities within New South Wales, AUS.  Exclusion criteria: Participants born overseas removed. | 1. Identify and establish the psychometric properties of a newly developed perceived discrimination and multiculturation (cultural respect) measure across a sample of Indigenous and non-Indigenous Australian secondary school students. 2. Determine if the measure is invariant across the sample of Indigenous and non-Indigenous students. 3. examine how PD and multiculturation may impact upon the subjective and objective schooling outcomes of Indigenous and non-Indigenous students, over and above the effects of socioeconomic home resources, gender, and the participants’ ratings of themselves as a student. | Quantitative | Cross-sectional survey administered in school halls under exam conditions. | The Perceived Discrimination and Multiculturation Scale (PDMS – Bodkin-Andrews et al., 2006). PDMS is a 13-item scale designed to unobtrusively measure agreement to experiencing perceived Personal Discrimination (e.g., *‘‘People have called me nasty names based on the culture I come from’’*), Macro Discrimination (e.g., *‘‘Other Australians don’t care about the hardships faced by people of my culture’’*), and Perceived Multiculturation (e.g., ‘‘*People I meet accept my cultural identity’*’) (1 = strongly disagree; 6 = strongly agree). | N/A | Academic disengagement; academic achievement; externalizing behaviour (self-report school absenteeism) | Invariance testing suggested that the PDMS may have equivalent meaning across the Indigenous and non-Indigenous students.  Indigenous students had significantly higher levels of Personal Discrimination (M = 2.7, SD = 1.34), and Macro Discrimination (M = 3.66, SD = 1.56), compared to non-Indigenous students (Personal Discrimination M = 1.87, SD = 1.02; Macro Discrimination M = 2.59, SD = 1.11).  The final model, with the inclusion of total discrimination, saw a significant improvement in model fit for both the Indigenous & non-Indigenous students. In this model, 9.7% of variance in Absenteeism for Indigenous students was explained, versus 2.5% variance explained for non-Indigenous students.  Discrimination (comprised of a 2^nd^-order factor score of Personal Discrimination and Macro-Discrimination) significantly predicted School Disengagement among Indigenous students (B = .36) and non-Indigenous students (B = .30), English achievement (Indigenous students: B = –.22; non-Indigenous: B = –.15), Math achievement (Indigenous students: B = –.23; Indigenous students: B = -.10), and Science achievement (Indigenous students: B = –.29; non-Indigenous students: B = –.10).  Multiculturation revealed no substantive impact on grades for either the Indigenous or non-Indigenous students |
| Bodkin-Andrews et al., (2013) | N = 1,376 (305 Indigenous;  1,071 non-Indigenous) students from 5 NSW high schools.  Exclusion criteria: Participants born overseas excluded from analysis. | 1) Once controlling for background variables and academic self-concept, how do multiculturation and discrimination impact on academic disengagement and self-sabotaging behaviour?  2) Is there an interaction effect between multiculturation and discrimination on academic disengagement and self-sabotaging  behaviour?  3) Do these findings differ across the Indigenous and non-Indigenous Australian students? | Quantitative | cross sectional, self-report survey (same as Bodkin-Andrews et al., 2010a; 2010b). | The Teacher Discrimination Scale was a five-item scale drawn from Wong et al.(2003) assessing the frequency to which students feel that teachers within their school have discriminated against them as a results of their race (e.g., *How often do you feel that teachers think you are less smart because of your race*?).  *Multiculturation* (perceived respect, acceptance, and pride of one’s own culture) | Proposed Multiculturation (respect for Indigenous perspectives in mainstream education) would enhance Indigenous youths’ ability to cope with discrimination. | Motivation and Engagement; academic disengagement; Self-sabotaging behaviours | Non-Indigenous students as compared with the Indigenous students reported higher academic self-concepts, and levels of respect for their culture, academic disengagement, and self-sabotaging.  Indigenous students reported experiencing more discrimination from their teachers (M = 1.84, SD = 1.04), compared to non-Indigenous students (M = 1.28, SD = .58).  Multiculturation had a positive effect on reducing the maladaptive outcomes for the non-Indigenous sample, yet had no impact on either of the outcomes for the Indigenous students  With the inclusion of teacher discrimination, significant portions of explained variance was added to the model for both the Indigenous and the non-Indigenous students across both self-sabotage (Indigenous: B = 0.40, p<.001, VE=16.4%; non-Indigenous: B =0.16, p<.001, VE=3.9%) and disengagement (Indigenous: B =0.30,p<.001, VE=12.1%; non-Indigenous: B =0.14,p<.001, VE=3.6%).  Significant interaction effect between multiculturation and discrimination  on self-sabotaging behaviour for the Indigenous participants. In the face of increased levels racism PD  from teachers, perceived multiculturation may exacerbate the risk of self-sabotaging behaviour. |
| Chen (2003) | Data collected in 1998-1999 on three American Indian  reservations in the Upper Midwest.  N = 212, 9-16 year old (M*age* girls = 12.1, M*age* boys = 12.2; 46% female). | Attempt to test "general strain theory" by tracing the linkage among the measures of PD, negative life events, family conflict, anger and depression, and early onset of substance abuse (i.e. will the same association between stressors and delinquent behaviour persist in this high-risk group?)  Detect moderating effects of social context and personal/social resources on association between stressors and negative emotional feelings, as well as the association between negative emotional feelings and delinquent behaviour. | Quantitative | Unclear if survey or interview. Switches between the two words. | Perceived discrimination scale validated with an Indigenous sample (Whitbeck, et al., 2001).  3 factor scale, example items include *threats of harm, hearing racial slurs* (global discrimination), *disrespect from shopkeeper, hassled by police because of ethnicity* (authority discrimination), and  *surprise at doing well at school, low expectations (*school discrimination). | Use of Tribal Advisory Board to inform study design. | Externalizing psychopathology (substance abuse), internalizing symptoms | Approximately half of the youths have been insulted by other kids (47.2%) or someone else yelled a racial slur or racial insult at them (50.0%). More than half of the adolescents (51.5%) reported that teachers were surprised when they did something really well.  Around one-quarter to one-third of these youths reported that they had been ignored by other kids, been treated unfairly, had teachers who had low expectations of them, been suspected by adults, or been treated disrespectfully by business. Around 10 to 15 percent of them reported that someone threatened to harm them physically or the police hassled them because they were American Indians.  PD construct, unexpectedly, was not significantly associated with substance abuse in this model.  Strong, positive association between PD and negative life events, as well as inconsistent parenting.  PD did not lead to higher levels of internalizing symptoms and use of alcohol and other illicit drugs, due to strong correlation between PD and other stressors, especially the experience of negative life events. |
| Quijada Cerecer (2013) | Data drawn from a 5-year qualitative study of Native American students who self-identified as Puebelo and attended a public school in New Mexico, US. Must have lived or currently live on a nearby reservation.  N = 21 (11 female, 10 male). | 1. How do school policies and rules affect adult-youth relationships for American Indian youth?  2. How do leadership practices affect adult-youth relationships and campus climate for American Indian students? | Qualitative | Focus groups; one-on-one interview; participant observation.  Data analysis involved counter storytelling methodological tools to identify themes that challenged the majoritarian story as told by Native youth. | environmental microaggressions/structural racism, overt racism | Used an Indigenous theory (TribCrit) to formulate research questions and analyse data. Use of storytelling methodology. | academic identity; cultural identity, educational development; and agency. | Two main themes: 1) structural racism was experienced by the youth through low expectations of achievement placed on students by teachers, as well as erasure of Indigenous Knowledges in curriculum, 2) students  identity was challenged and opportunities for expressing autonomy were supressed by the introduction of a school dress code and police officer. |
| Davis et al., (2019) | Participants recruited from 31 schools in the US (except for Oklahoma) as part of an ongoing epidemiologic investigation of substance use among Native American youth. Random sampling of schools. Youth who had reported drinking in their lifetime included in analyses.  Inclusion criteria: in grades 7–12, live on or near reservations. N = 1,934 (M*age* = 15.31; 55% female, 1% gender not stated). | To explore classes of drinking motives (drinking to cope, drinking for pleasure) as they relate to heavy episodic drinking, perceived discrimination, religious importance, ethnic identity, and ethnic pride. | Quantitative | Participants completed the Our Youth, Our Future (OYOF) survey online.  Latent profile analysis to identify to identify classes of youth who drink based on their responses to drinking motives survey items. | PD measured via Whitbeck et al., (2001) scale. | Examined Ethnic Pride and Ethnic Identity as covariates. | Externalizing behaviour (alcohol use); coping. | Approximately 69% (n = 1,326) of the sample reported not having consumed five or more drinks in one sitting within the last two weeks.  2-class model had best fit. Class 1 characterized by high endorsement of drinking to cope and high endorsement of drinking to enhance positive affect. Class 2 characterized by low endorsement of drinking to cope and drinking for enhancement of positive affect. Class structure consistent across age groups. Class 1 reported significantly higher levels of PD, as well as drinking episodes. |
| Dickerson et al., (2019) | Participants recruited participants as part of RCT of Motivational Interviewing and Culture for Urban Native American Youth, California, US.  Inclusion criteria:  verbally self-identifying as Native American (or being verbally identified by a family or community member). Aged 14–18.  N = 182 (M*age* = 15.6, 52% female). | Examine associations between perceived overt discrimination racial microaggressions (RMA) and health outcomes.   1. Does participation in traditional practice moderate relations between PD, RMA and health outcomes? | Quantitative | Self- report, cross-sectional survey.  Association between overt PD and RMA and health outcomes assed via linear and logistic regression. Odds ratios reported for the effects of experiencing five additional overt PD/RMA events, the interquartile range of overt PD/RMA events in the sample. | Experiences with both overt PD and RMA were measured using the short version of the Microaggressions Distress Scale (Walters, 2009). Ten items asked about verbal, behavioural and environmental encounters in the last year that implicitly or explicitly diminished racial heritage, identity, or culture (e.g., asked if real Indian by non-natives). | Data came from RCT of Motivational Interviewing intervention that was developed with AI/AN traditional healing practices, culturally appropriate recruitment methods (e.g., at Pow Wow, community events, community forums, organisations serving AI/AN youth), included Traditional Practices as a covariate | Externalizing behaviours (referred to by authors as 'health risk behaviours'; heavy drinking, marijuana use, tobacco use, consequences of substance use). Mental Health (depression and anxiety symptoms over last month); General Health Status. | Adolescents reported experiencing a mean of 3.57 (SD = 2.64) overt PD/RMA events in the past year, with a 25th percentile of 1, a median of 3.5, and a75th percentile of 6. Thirty-two adolescents (18%) reported no overt PD/RMA in the past year, and an additional 19 (10%) reported only one form of overt PD/RMA. Thirty-one (17%) reported at least seven of the ten forms of overt PD/RMA. The remaining 55% experienced between two and six forms of overt PD/RMA.  Adolescents reported having participated in an average (SD) of 95.1 (97.6) traditional practices.  After adjusting for age and gender, each additional five overt PD/RMA events were associated with 2.49 times the odds of reporting cigarette use in the past year. Smaller non-significant associations and similarly wide confidence intervals were found for nearly all substance use outcomes after adjusting for age and gender: e-cigarette use in the past year.  There was a strong and significant association between overt PD/RMA and alcohol-related consequences in the past 3 months (AOR 3.02).  Past month mental health sores were estimated to be 3.31 units lower for every five additional events after adjusting for age and gender, but the confidence interval was large, and included every-thing from an 8.38-unit deficit to a 1.76-unit benefit for the high-overt PD/RMA adolescents.  Due to the low numbers of youth who did not engage in traditional practices (3.3%), we were unable to analyse whether effects of overt PD and RMA on health behaviours were moderated by traditional activity participation. |
| Edwards-Groves (2008) | N = 17 Australian Aboriginal boys.  Data for this study were collected with male Aboriginal adolescents enrolled at Tirkandi. The centre is an intervention initiative for young Aboriginal boys who show potential but are at risk of entering the criminal justice system.  Participants come from New South Wales. The sample of participants was small and non-representative of all Aboriginal youth's perspective of schooling. | Explore perceptions of school experiences by male Aboriginal youth at risk of becoming in contact with the juvenile justice system. | Qualitative | At 12 different sessions, interviews were conducted with 17 boys. Data was collected during semi-structured tape recorded.  Individual "photo-interviews" with 10 participants, focus group "photo-interviews" with three participants, informal discussions with groups of students during participant observation sessions in classrooms, leisure activities and cultural programs. | Racism at school. | Photovoice methodology using cultural items important to the youth participants. | N/A | Racism was identified as a significant issue when participants were asked about challenges faced at school.  A 14 year-old boy gave an evocative account of what this boy takes racism to be "when people don't respect you, they swear at you and make fun of you because you are 'black'; ‘ because you are Aboriginal; people swearing at me all the time, fighting with me".  In the accounts from these boys, the signs are everywhere that racism is experienced within the interactive experiences of these people in their day-to-day lives at school.  “I feel stuck in the middle sometimes, I am too white to be black, and too black to be white”.  Other manifestations of racism included being ignored by teachers, getting incorrectly blamed for misconduct and not being trusted or provided with responsibility.  Racism in the classroom lead to lack of agency at school overtime. |
| Garrett, et al., (2017). | Data were part of a group-randomized controlled trial to pre-vent alcohol use among youths.  N = 1421 White, White/American Indian and American Indian youth, 14-19 years.  The sample was 49.8% male and primarily low income (56.9%), with a mean age of 15.9. | Hypothesized that perceived racial discrimination would predict the use of substances. | Quantitative | Two self-report surveys approximately six months apart. The sample was drawn from the baseline period of the prevention trial prior to the implementation of the intervention. | Frequency of perceived discrimination and intensity of discrimination experiences were assessed by two items drawn from the National Youth Risk Behaviour Survey(Centres for Disease Control and Prevention, 2010). “*How often have you experienced any kind of discrimination due to your* race/ethnicity?”  Response options were “never,” “hardly ever,” “a few times a year,”  “monthly” and “daily.”  Intensity of discrimination was assessed by asking *“How would you describe the discrimination you have experienced?*  Response options were “I have not experienced any kind of discrimination due to my race/ethnicity”; not very disturbing”; “somewhat disturbing”; or “very disturbing.” | Cherokee Nation approved data collection procedures, protocols and analyses. | Externalizing behaviours (substance use, tobacco use) | ‘No’ discrimination reported by 74.4% of AI participants, 73.4% of AI/White participants, and 82,3% of White participants.  23.8% of AI participants reported ‘moderate’ discrimination (23.6% for AI/White participants; 15.4% for White participants).  1.8%o of AI participants reported ‘high’ discrimination (3% AI/White participants; 2% White participants).  Majority of AI, AI/White, and White participants reported discrimination as ‘not very disturbing’. 6.36% of AI youth reported discrimination as ‘very disturbing’ (2.3% AI/White; 3.8% White).    Race/ethnicity did not moderate the effects of frequency and intensity of perceived racial discrimination on sub-stance use outcomes. Given this, the results were presented for the combined sample. |
| Greenfield, et al. (2017). | Participants from 3 Indigenous Reservations in the northern Midwest and 4 Canadian Reserves. Data from one Reservation are not included due to community request.  Inclusion criteria: Each of the participating reservations and reserves provided a list of families of tribally enrolled individuals aged 10 to 12 years who lived on or within 50 miles of the reservation or reserve.  N = 673 adolescents who completed baseline (Wave 1) diagnostic interviews (M*age* =11.1, SD = 0.82; 50.3% female). Approximately 11% lived in a remote location (compared to a rural location), and 85.5% lived on a reservation/reserve. | Examined joint trajectories of Conduct Disorder (CD) D and Alcohol Use Disorder (AUD) or lack thereof—among Indigenous individuals aged 10 to 18 years. | Quantitative | Longitudinal survey.  Eight waves of data collected with the adolescent and at least one primary caretaker. via yearly interviews. Study analysis used Waves 1, 4, 6, and 8, when diagnostic data were collected, and aligned participants by their age at each wave.  Group-based trajectory modelling to determine trajectory groups of comorbid Conduct Disorder (CD) and Alcohol Use Disorder (AUD), to identify risk factors that prospectively predict trajectory group membership, and to characterize their associated age-18 outcomes. | PD assessed with 10 items from an adapted version of the Schedule of Racist Events, measuring how often adolescents experienced negative treatment from others because of their indigenous culture. | Study team had a research partnership with northern Midwest and Candian reservations. Team worked on the reservation and tribal resolutions obtained. Respected on reservations request to keep data private. | Externalizing psychopathology (CD, AUD), school completion; teenage pregnancy; delinquent behaviour. | Participants experienced an average of .31 PD events (max = 1.7 events).  Majority of participants did not develop CD or AUD.  The high CD with later-onset AUD group had significantly higher PD (average of .60 discrimination events) than the asymptomatic group (average of .27 discrimination events) Membership in high CD later-onset AUD group significantly increased the odds of engaging in sex under the influence (OR = 4.34). |
| Harre & Pidgeon (2011) | N = 39 Anishinaabe youth, ages 16-20, on-reserve communities in northern Ontario. | To identify the inequalities Indigenous youth faced in their schooling and how they exercised strategies of agency and resistance to allow them to take a warrior’s stance…that allowed them to reframe their educational experiences” | Qualitative | Open ended questions that were audio-recorded, sometimes video-recorded too | Structural. | Utilised Kanein’kehaka (Mohawk) scholar, Taiaiake Alfred (2005)’s concept of ‘new warrior’ to frame investigation of First Nation Canadian youths’ school experiences. | Coping; educational engagement. | 3 distinct themes;   1. Youth experienced different forms of racism and discrimination, which were pervasive and perpetuated by teachers, peers and the school curriculum. 2. The pivotal role that family played, immediate and extended, in supporting and strengthening Indigenous community values that enabled Anishinnaabe youth to persist in schooling. 3. The availability of alternative school options, located in First Nation communities and controlled by them, for those students who resisted mainstream public-schooling. |
| Hartshorn et al., (2012) | Data collected as part of ongoing longitudinal study designed in partnership with four Canadian First Nation Reserves.  N = 692 Indigenous adolescents (mean age = 12 years) from the Northern Midwest of the United States and Canada. Rural sample. | Examined the proximal and distal effects of PD on adolescent aggression.  1) Does early aggression lead to PD, or do perceptions of discrimination lead to aggressive behaviour?  2) Does aggressive behaviour precede adolescent anger, or does anger lead to aggression?  3) Does anger mediate the association between PD and aggression over time. | Quantitative | Longitudinal, self-report survey.  Autoregressive cross-lagged path analysis. Age, gender, remote location, and per capita family income were controlled for as part of the method. | Perceived discrimination via adapted version of the SRE. This measure is a mean indicator of the frequency of experiencing specific instances of dis-crimination questions regarding how often in the past 12 months the following experiences occurred because of their ethnicity. | Ongoing longitudinal study designed in partnership with four US reservations and four Canadian First Nations reserves, use of Tribal Advisory Council who handles personnel difficulties, advised on questionnaire development, reviewed and approved reports and presentations. Used measure of discrimination adapted for First Nations groups. | Externalizing symptoms (aggression); anger | PD significantly and positively associated with later aggression (even when controlling for prior aggression, age, gender, incomes and location.  Anger positively associated with increases in aggression (controlling for covariates).  Relation between prior PD and future aggression was partially mediated by anger. |
| Hopkins et al., (2014) | A subset of 1,021 youth aged 12-17 derived from the Western Australian Aboriginal Child Health Survey (WAACHS 2000-2002).  WAACHS is a population representative survey of 0-17 year old Aboriginal children, their family and community contexts. | To identify the factors that uniquely protect psychosocial development within high and low family-risk exposed 12–17 year old Western Australian Aboriginal youth. | Quantitative | Survey, parent/carer report, self-report.  Multivariate logistic regression was used to model the influence of individual, family, cultural and community factors on psychosocial outcomes separately for youth in high and low family-risk contexts. | Youth self-reported response (1 =no, 2= yes) to a single item asking whether, *‘‘in the past 6 months, have you ever been treated badly or refused service because you are Aboriginal?’’* | Sought ethics approval from local Aboriginal ethics committee. Included youth cultural knowledge, speaks Aboriginal language, importance of ceremonial business as covariates. These factors were not independently associated with psychosocial functioning. | Psychosocial functioning | For youth in high family- level risk contexts, having a prosocial friend conferred unique protection (p=.02, OR 2.57, 95% CI 1.17, 5.64), and living in more socioeconomically advantaged neighbourhoods conferred additional risk.  Relative to youth in the lowest 10% of neighbourhoods ranked by socioeconomic advantage, those youth in the highest 50% of neighbourhoods were less likely to be Resilient (OR .42,).  In low family-risk contexts, youth reporting not being exposed to racism were more than twice as likely (OR 2.09) as those exposed to racism to have good psychosocial functioning.  Finally, two factors at the individual level were identified as generally beneficial for Aboriginal youth in both high and low family risk exposed contexts. Higher levels of self-esteem and self- regulation (no reported fighting in the last 6 months) were significantly associated with normal psychosocial functioning for both Resilient (high family risk) and Expected Good (low family risk) youth. |
| Jaramillo et al., (2016) | Data were collected from schools in 2009.  N = 129 individuals who self-identified as Native American. M*age* = 16.38 (S.D = 1.15, Range 14-19 years) Almost 50% of the sample was female, low socioeconomic status. | 1) Is ethnic identity related to academic achievement and hopelessness?  2) Does stereotype threat and PD relate to academic achievement and hopelessness?  3) Does ethnic identity moderate the relationship between stereotype threat or PD and academic achievement or hopelessness? | Quantitative | Cross-sectional, self-report survey.  Multiple regression analyses. | The Perceived Discrimination Measure (Whitbeck, et al., 2001) | Included ethnic identity as protective factor. | Ethnic identity; academic achievement; hopelessness | Average PD score for the sample was 1.84 (SD = .63, min = 1.00, max = 3.7).  Ethnic identity and PD not related to academic achievement and or hopelessness. The only meaningful correlation was a positive association between stereotype threat and hopelessness (r = .34, p < .001).  Ethnic identity, stereotype threat, and their interaction predicted academic achievement, F(5,120) = 2.11, p < .01). This model explained 4% of the variance in academic achievement. Simple slopes analyses indicated the slope for low ethnic identity was not significant, b = .06, t(126) = 0.82, p = .42, whereas the slope for high ethnic identity was significant, b = .14, t(126) = 2.05, p < .05. High ethnic identity youth had a significant positive relation between stereotype threat and academic achievement.  Hopelessness was predicted by ethnic stereotype threat, PD, and their interactions. This model explained 13% of the variance in hopelessness, F(6, 119) = 4.88, p < .001). Ethnic identity interacted with PD to predict hopelessness, where participants with low ethnic identity and high PD scores were higher in hopelessness.  Findings suggest that it is the joint process of identification with one’s ethnic group and the awareness of the prejudices associated with that group that are associated with academic and psychological outcomes |
| Johnston-Goodstar & Roholt (2017) | Inclusion criteria: self-identifying as Native American or as having a professional capacity with Native American young people.  This article reports on study data relevant to education and schools, including field observation, interviews conducted with youth workers, educators, elders, and community members from our tribal partners and state-wide  Native American community members (N= 42).  An independent analysis of the 2010 Minnesota Student Survey typically completed by students in all schools across the state (N= 6,840 Native American youth)  Targeted surveying of state wide school policy and discipline records (N= 12)  Youth focus group participants (N= 53), and a review of secondary qualitative data held by the Minnesota Historical Society (N=3). | The study sought to challenge this individualized deficit analysis of school success by asking questions such as how are Native youths experiencing schools, what messages are they receiving in school and in society, and how do these experiences and messages impact their school success? | Qualitative and quantitative | Community-based participatory research (CBPR) collaboration with a Midwestern tribal community, that utilised community mixed methods. Interviews were conducted in teams, using a semi-structured interview format.  All data from field observations, interviews, youth focus groups, and secondary qualitative data were entered into an electronic qualitative data analysis platform. | microaggressions | Community-based participatory research (CBPR). | academic retention. | Microassaults were observed on social media, at sporting events, during interpersonal interactions, and in disciplinary patterns. and included discrimination at school sporting events, discrimination in the school. discrimination in discipline  Microinsults including feelings of *Invisibility*, *Tracking and Labelling, and Assumptions of deficiency and pathology*  Microinvalidations included  *Invalidating historical trauma,*  *Invalidating native voices* and  *Invalidating sovereignty*  Seventy-seven percent of our youth focus group participants reported being “called a racial slur at school, “with 37% reporting this slur came from a “non-Native” student,”29%reporting the slur came from a “teacher or school staff, “and 8% reporting the slur came from a “non-Native” adult in the community. |
| Galliher et al. (2011) | Participants recruited through schools.  Inclusion criteria: English fluent, ages 14-19 at Wave 1.  Wave 1: n = 137. Navajo students (67 male/70 female) (median age = 15), year 9 (62%), year 10 (28.5), year 11 (5.1%), year 12 (2.9%).  Wave 2: n = 92. Navajo students (41 male/51 female), ages 16-21 (median = 17), year 10 (6.5%), year 11 (62.%), year 12 (24%). | 1. How do reported ethnic identity and experiences of discrimination change over  a 2-year period in high school?  2. In what ways are the development of ethnic identity and experiences of  discrimination related to each other over time?  3. In what ways do reported ethnic identity and experiences of discrimination  influence psychosocial outcomes (i.e., depression, self-esteem, social functioning, school  achievement, school connectedness, delinquent behaviours, and substance use) of Navajo  adolescents both concurrently and over time? | Quantitative | Self-report survey – longitudinal.  MANOVAs to test for gender differences across study variables (Belonging  and Exploration, OCIS American Indian and White American, Discrimination, Self-esteem, Social Functioning, and Substance Use) | Measured by Yoder (2001) Discrimination scale. | Included ethnic identification as a covariate/resiliency factor, sought study approval from the Navajo Nation Human Research Review Board. | Self-esteem, belonging and exploration, social functioning, externalizing behaviour (substance use) | Wave 1 average discrimination scores: Male = 1.67 (SD = 0.57, max possible score = 4), Female = M = 1.56, (SD = 0.48).  Wave 2 average discrimination scores: Male = 1.78 (SD = 0.70), Female = 1.52 (SD = 0.48). Males experienced significantly higher discrimination than females at Wave 1 but not Wave 2. Males reported significantly higher discrimination from Wave 1 to Wave 2. No effect sizes noted.  Wave 1 discrimination experiences were linked to W1 lower self-esteem (B = -.311) and social functioning (B = -.295) for male adolescents.  Perceptions of discrimination serve as a major risk factor for Navajo adolescents, especially male adolescents.  Male participants reported significant increases in Discrimination from W1 to W2. A very consistent, strong relation emerged for male adolescents between discrimination experiences at Time 1 and substance use frequency at Time 2.  For males: 1) W2 interactions:  few significant direct longitudinal effects of ethnic identification variables emerged, above and beyond the variability accounted for by Time 1 psychosocial functioning scores.  At the highest levels of American Indian identification, a positive relation emerged between discrimination and social functioning, suggesting a counterintuitive longitudinal effect of discrimination for those who report the highest level of engagement and involvement in American Indian culture.  A strong positive relation between discrimination experiences and substance use for those with the highest levels of White American identification and, basically, no relation between discrimination scores and substance use for those at the lowest levels of White American identification.  For females: a positive relation between discrimination experiences and self-esteem emerged for girls at the highest level of White American identification, whereas a negative relation be-tween discrimination and self-esteem was observed for girls at the lowest levels of White American identification.  One reason for female adolescents’ consistency in positive outcomes over time and overall more positive functioning may be rooted in matriarchal Navajo culture  A tribal partnership with researchers focusing on establishing and promoting the role of Navajo men and women may be necessary to combat the negative influences brought about by intergenerational trauma. |
| Martinez & Armenta (2020) | First Nations people from three U.S. American  Indian Reservations and four Canadian First Nations Reserves. The participating reservations and reserves share a common cultural tradition and  language with only minor regional variations.    Wave 1 N = 674 adolescents  (M*age* = 11.10, SD = 0.83; 50.3% girls), of which  94.7% completed Wave 2 (50.1% girls), 92.9% completed  Wave 3 (49.8% girls), 87.3% completed Wave  4 (50.8% girls), 87.7% completed Wave 6 (50.3%  girls), and 77.6% completed Wave 8 (52.9% girls). | 1. to identify the distinct developmental patterns  of depressive symptoms that our sample of Indigenous  youths followed as they progressed through  adolescence.  2. to consider  whether membership in the depressive symptoms  trajectory groups varied as a function of personal,  familial, and minority-related factors. | Quantitative | Longitudinal self-report survey | Modified version of the SRE. | Community partnerships and Tribal Advisory Boards | Psychopathology (depression) | 1. Four depressive symptoms trajectories were identified: (a) sustained low, (b) initially low but increasing, (c) initially high but decreasing, and (d)  sustained high levels of depressive symptoms.  2. Trajectory group membership varied as a function of gender, pubertal development, caregiver major depression, and PD  3. higher levels of PD increased the risk for being in the high-decreasing and high depressive symptoms  groups, relative to the low and low-increasing depressive symptoms groups.  4. participants in the different trajectory groups were at differential risk for the development of an alcohol use disorder - high-decreasing and high depressive symptoms groups at greatest risk, relative to other groups. |
| MacDonald (2019) | N = 12 (4 high-achieving Māori  female students, 16 & 17 years of age), 1 parent per student (n = 4), 1 friend per student (n = 4). Not representative. Gender of parents/friends unspecified. | To examine how the lived experiences of four high-achieving, Māori girls  from one mainstream school navigate ‘speaks back’ to a hidden curriculum of settler  silencing. | Qualitative | semi-structured, in-depth interviews. Data thematically analysed and framed by critical race theory. | Structural racism, internalised racism | Interviews guided by Kaupapa Maori methodologies, included member checking of data. | identity, educational attainment | Silencing through cultural and ethnic approaches construct indigenous youth identities in ways that maintain white normativity, particularly as they strive to be academically successful.  Students must overcome significant barriers and make personal concessions to accomplish Eurocentric notions of schooling success.  Students’ resilient behaviour to silencing and their ability to speak back to the hidden curriculum must be considered in relation to other ways they are socially  positioned.  Enormous challenges faced by Māori students who are differently socially positioned and striving for academic success. |
| Melander et al., (2013) | Participants were First Nations people (from 4 US reserves, 4 Canadian reserves). Selected reservations/reserves share a common cultural tradition and language with minor regional variations in dialects. Sample represents one the most populous Indigenous cultures in the U.S. and Canada.  N = 702 (50% male), aged 11-14 (M*age*= 12 years). | To examine the relationship between familial, educational,  and psychosocial factors and bullying among North American Indigenous adolescents | Quantitative | Longitudinal self-report survey.  ANOVA's, Scheffes post-hoc test, Chi-Square tests (e.g. IV's parental warmth, perceived discrimination) DV - bullying status (Perpetrator/Victim/Neither). Multivariate analysis - Multinomial logistic regression to predict bullying status using the same covariates from the bivariate analysis e.g. PD. | Adapted from the SRE. | Ongoing longitudinal study designed in partnership with four US reservations and four Canadian First Nations reserves, use of Tribal Council-appointed advisory boards who handled personnel difficulties, advised on questionnaire development. | Externalizing behaviour (bullying), educational attainment | Mean score for PD for sample was .019 (SD = .32, range = 0-3).  Participants who were neither bullies or victims had the lowest levels of PD (mean = .11). Participants who were both victims and perpetrators had higher levels of PD (mean = .44) compared to participants who were just perpetrators (mean = .26).  As hypothesized, anger and PD were associated with being a bully perpetrator. Relative to being “neither” the odds of being a bully increased by 376% for each unit increase in anger (B = 1.56) and by 396% for each unit increase in PD (B = 1.60).  Relative to being neither a bully nor a victim, the odds of being a victim increased by 7% for each unit increase in depressive symptoms( B = 0.07) and by 563% for each unit increase in PD (B = 1.89). PD associated with increased odds of being a bully/victim.  Several factors differentiated being a bully perpetrator from being a bully victim: adolescent age, parental warmth and support, depressive symptoms, anger, and school adjustment. |
| Middlebrook (2010) | N = 410 adolescents (31% American Indian, 69% European American). 14 - 19 years old (median age = 16.09 years; 54% female). AI sample 50% female.  The AI participants came entirely from a rural AI governed school on the reservation, and a rural school in a town which borders the reservation. The European-American participants came from the rural border town school in the Mountain state, as well as from urban schools in a Western state. | Investigate the nature of the relationship among social identity variables, attitudinal variables and academic achievement of AI students.  1.Are there differences between AI students’ and European-American students’ ethnic identity, school identification, perceived barriers, PD, and educational utility?  2.Does ethnic identity contribute to the variance in AI's achievement and school identification beyond perceived barriers, PD, and perceived educational utility?  3. Do meaningful groups based on levels of ethnic identity and school identification in AI populations exist? | Quantitative | Self- report cross- sectional survey.  Cluster analysis was performed in order to see if meaningful groups of AI participants based on levels of ethnic identity and school identification could be identified. Six additional t-tests were conducted in order to see if the clusters differed on the basis of GPA, perceived discrimination, perceived barriers, and concrete, abstract, and ambivalent educational utility. | McWhirter’s (1997) Perceived Barriers. The ethnic discrimination subscale consists of 4-items which refer to perceived barriers to a professional career due to ethnicity. Participants respond to the items on a 5-point Likert scale that ranges from 1 (strongly agree) to 5 (strongly disagree). E.g., *“In my future job, I will probably be treated differently because of my ethnicity”*  PD assessed via Whitbeck et al., (2001) scale. | Included measure of ethnic identification. | Academic achievement; school identification. | Significant differences between AI and EA students on 8 of 9 variables (all with large effect sizes): AI participants reported higher scores on measures of abstract, concrete, combined, and ambivalent educational utility, as well as on measures of ethnic identity, perceived barriers, and perceived discrimination The EA participants had significantly higher GPAs.  Mean PD scores: AI = 1.84 (SD = .62), EA = 1.33 (SD = .58), significant difference.    Mean Perceived Barriers scores: AI = 2.4 (SD = .98), EA = 1.68 (.78). significant difference.  Cluster 1 = Low School Identification. Cluster 2 = High School Identification. No statistically significant differences were found between the two clusters on GPA, perceived discrimination, perceived barriers, or abstract, ambivalent, and concrete educational utility.  PD not associated with school identification. The findings that the two clusters did not differ on measures of educational utility, GPA, perceived barriers, or perceived discrimination calls into question the usefulness of clustering this AI sample based on their school identification. It is possible that within the context of a homogeneous, tribally operated school, school identification has less of a relationship with attitudes and behaviours than it would in a more diverse school setting. |
| Nelson, & Hay (2010) | Participants recruited as part of a larger study on physical activity.  N = 14 Aboriginal and Torres Strait Islander  adolescents aged 11-15 years. (6 male, 8 female). Urban, enrolled in a non-government school with a large number of Indigenous students. | To explore Indigenous students experiences of school and to convey a sense of the varied and complex nature of their educational and life pathways. | Qualitative | Interview. Each participant interviewed seven times across 2.5 years as they transitioned from primary to secondary school. | Overt, covert. | Participants given their data back via a “digital story” and researchers sought their permission to represent this with this content in academic publications. | N/A | Moving Schools: 10/14 students moved schools during research period, all reported maintaining strong connection to their Indigeneity. (2) Factors influencing perceptions of school and decision to remain at particular school: flexibility of school in catering to students changes in life circumstance and needs, strong relationships between student/family and school/school staff, perception of school’s behaviour management approach, and academic opportunities. (3) Future aspirations: diversity in aspirations of participants. (4) Students generally positive about future pathways, saw Indigeneity as one of their key strengths.  One participant reported being called ‘White’ by other Inidgneous students due to their lighter skin colour.  Several students had experienced racism. For example, “people say bad things about my culture and all that". This had occurred "around the shops" when people had said "watch out you black..”  Participant was also aware of public stereotypes about her as an Aboriginal young woman, stating that she thought it was important that people knew that Indigenous young people "are at school and they do you know work". Another participant said she did not feel sae and she had experienced racism at her new school and she noted with wry humour that "I get called Blackie at school". |
| Priest et al (2019). | N = 2802 (19.3% visible ethnic minority non-white, non-Indigenous, 2.6% Indigenous).  49.14% of sample female,  NOTE: sample was split by Indigenous status only for descriptive statistics. | (1) What are the patterns of cumulative exposure to bullying victimization and PD, separately and combined, among Australian adolescents across Indigenous and ethnic background?  (2) What is the impact of cumulative exposure to bullying victimization and PD on social and emotional development, BMI z-score, and over- weight/obesity among Australian adolescents? | Quantitative | Data drawn from Longitudinal Study of Australian Children (LSAC) - Australian Bureau of Statistics  Data begins when subjects are 10-11 years old and ends when they were 14-15. | Perceived discrimination.  Children were asked whether in the last six months they had been treated unfairly or badly because of their language or accent, skin colour, or cultural background. | N/A | Socioemotional difficulties were measured at W4 and W6 using the “total difficulties score” from the parent-reported Strengths and Difficulties Questionnaire (SDQ; Goodman and Goodman 2009). | Indigenous participants had been bullied at one time point (either W5 or W6) and over a quarter (27%) that they had been bullied in both W5 and W6. Racial discrimination was reported by just over a fifth (21%) of Indigenous children at one time point and by 10% at both time points.  Combined exposure to both bullying victimization and racial discrimination was also highest among Indigenous children, followed by visible minority children.  Regression models predicting SDQ scores did not separate Indigenous youth from ethnic minority or non-Indigenous youth.  NOTE: Authors note that “poxy ethnicity categories that identify stigmatized identities based on parental country of birth and Indigenous status were created: Australian-born, Anglo/European (Caucasian or white), visible minority (non-Caucasian or non-white, not Indigenous), or Indigenous” although this categorization incorrectly delineates Indigenous youth as not being born in Australia. |
| Priest et al., (2016) | Aboriginal and Torres Strait Islander,  Non-Indigenous Australians, ethnic minority Australians  Data from 3956 children aged 12 to 13 years from wave 5 (2011–2012) of the nationally representative (LSAC) kindergarten cohort. | To compare the prevalence of bullying victimization and racial discrimination by ethnicity. | Quantitative | Data drawn from Longitudinal Study of Australian Children (LSAC) - Australian Bureau of Statistics, Survey, self and parent report. | Children were asked 3 questions about racial discrimination experiences developed for LSAC from which we derived a single dichotomous variable. | N/A | N/A | Compared with children with Australian-born parents, Indigenous children reported the highest levels of physical bullying (44.4% vs 31.8% ;crude prevalence rate ratio = 1.40; 95%CI = 1.11, 1.76), social bullying (46.6% vs34.3%; crude prevalence rate ratio = 1.36;95% CI = 1.06, 1.74), any bullying (69.1% vs55.7%; crude prevalence rate ratio = 1.24;95% CI = 1.07, 1.43), and racial discrimination (23.0% vs 8.8%; crude prevalence rate ratio = 2.63; 95% CI = 1.76, 3.94).  Concordance between the any bullying and racial discrimination measures was poor among all ethnic groups (Indigenous: 49.5% ;k= 0.11), suggesting they are distinct stressors in children’s lives. |
| Priest et al., (2011) | Data from pre-existing dataset, included Aboriginal and Torres Strait Islander  participants who lived in in the Northern Territory, Australia.  N = 345, aged 16-20 (Mage = 18.27 years). Gender not noted. | To explore the associations between self-reported racism and health and wellbeing outcomes for young Aboriginal Australian people. | Quantitative | Data drawn from Government data - Aboriginal Birth Cohort Study. | Participants were asked “*Have you been treated unfairly or discriminated against because you are Aboriginal?”* and were asked to select one of three responses: little bit, fair bit or lots. Data were recoded into two groups for analysis (“little bit” and “fair bit/lots”). | Sought approval for the study from the Aboriginal Ethics Subcommittee, assessed Aboriginal and Torres Strait Islander specified outcomes (i.e., social emotional wellbeing outcomes) | Psychopathology (anxiety, depression); suicide risk; overall mental health | Racism self-reported by 32% of participants. Racism was significantly associated with anxiety, depression, suicide risk and poor overall mental health.  No significant associations were found between self-reported racism and resilience. |
| Sahdra et al., (2020) | N= 898 high school student participants. Mage = 14.13 years. 46.2% female, 52% male, 1.7% other).  46% White, 20% Australian Indigenous, 34% other minorities. | To understand if youth feel better or worse to the extent that they feel less or more discrimination than their friends. To what extent is the frame of reference of overlapping friendship communities important for young people’s feelings of discrimination and subjective wellbeing? | Quantitative. | Self-report, cross-sectional survey  Multilevel models to examine ethnic differences in terms of the psychological variables. | Perceived discrimination.  Adapted a measure from King et al. (2007). The m*easure consisted of the following four items: “I have been discriminated against in education because of my cultural background,” “Sometimes I feel that I am being talked down to because of my cultural background,” “I have been discriminated against by the police because of my cultural background,” “I would have had better chances in life if I had come from a different cultural background.”* Disagree to 5 = Strongly Agree). | N/A | Mental ill-health symptoms within the last 12 months; Subjective wellbeing. | Relative to Whites, Indigenous and other minority Australians reported greater discrimination against their group, but the three ethnic groups were comparable in terms of mental ill-health and subjective wellbeing, though there was a tendency for Indigenous and other ethnic minorities youth to report greater wellbeing than their White counterparts.  ‘Frame of reference’ analysis not broken down by Indigenous status. |
| Schinke et al., (2010). | Wikwemikong (Canadian Indigenous Community – Unceded Indian Reserve) athletes.  Phase 1 - N=30 community members (2 x n = 15Talking Circles).  Phase 2:  5 Adolescent Talking Circles, N= 40 – 50 Indigenous adolescents (8-10 participants per Talking Circle). | Understand  the adaptation experiences (challenges and strategies) of reserve youth when they travel to compete in sporting events off reserve (“mainstream”). | Qualitative | 2 initial Talking Circles with Indigenous community members on reserve to share their thoughts on adolescent participation in athletics.  5 additional Talking Circles with Indigenous adolescents on the reserve.  Talk in circle and 1 by 1 answer questions, holding stone to show they are speaking. Led by community coresearcher.  Questions about activities what enables and gets in the way of participation; what the benefits and down sides of activity participation are; experiences of cross-cultural discrimination in sports.  Community-led thematic analysis of Talking Circle transcripts. Final codes were reviewed and amended by Community Leaders. | Does not distinguish between forms – assumption is overt, however. E.g. racist slurs etc. | Talking Circles. Community members part of data analysis and interpretation. | Coping (i.e., responses to racism and discrimination. | Participants’ challenges in sport included racism via) political agendas privileging local ‘mainstream’ athletes for sporting teams, racist opponents, including slurs, physical altercations, throwing things at team from crowd, mainstream admin ignoring racism and being racist.  Participants engaged in both coping responses to racism: either via Maano (passive responses) form of power in ignoring racism, avoiding racist people/situations “leave it alone” or via Active response – educate, “talk back”, challenge racism. Both coping responses viewed as adaptive.  Trust also important, as adolescents trusted family and community to support them consistently.  Belonging evident in wanting to positively represent Wikwemikong & fear of misrepresentation.  Significance of extending developmental (adaptation) theories though culturally responsive and reflexive research practices for Indigenous and other communities. |
| Walls, et al., (2016). | Participants recruited from 4 American Indian Reserves and  4 Canadian Native American Reserves  N =  569 Indigenous adolescents (M*age* =17.23; 51% girls)  N = 563 Indigenous adult caregivers (M*age* = 44.66; 77.4% women). | (1) Does endorsement of traditional Indigenous spirituality offer protective effects for mental health?  (2) Do culturally relevant stressors increase mental distress?  (3) What is the interplay of culturally specific risk and resiliency factors in relation to mental health outcomes? | Quantitative | Survey administered via interview with youth and care-taker.  Measures:  1) Traditional spiritual activities (both samples)  2) Perceived discrimination (both samples – but slightly different measures)  3) Historical loss (both samples)  4) Psychological well-being/distress (both samples – slightly different measures)  Descriptive estimates of zero-order correlations – then 4-latent variable regression models. | PD assessed via 12 items modified from the SRE. | Study designed in partnership with communities, Tribal Advisory Boards | Internalizing psychopathology (depressive symptoms, anxiety), anger. | When culturally relevant risk factors were included in multivariate modelling, the impact of spirituality on psychosocial outcomes dropped from statistical significance.  Very important to consider Indigenous cultural practices (spirituality) in the context of cultural risk factors such as discrimination and loss; because otherwise can lead to false correlation between TSA and worse mental health.  Mean perceived discrimination in adolescent sample = .18 (SD =.25, scale range 0-2).  In adolescent sample: The traditional spiritual activity measure was positively and significantly associated with perceived discrimination and historical loss (moderate effect size). The traditional spiritual activities, perceived discrimination, and historical loss measures each were positively and significantly associated with depressive symptoms, anxiety, and anger (small to moderate effect sizes).  TSA positively associated with depressive symptoms, anxiety and anger; however, this became nonsignificant when the perceived discrimination, historical loss or both were added as additional predictors.  In the final model (Model 4), both perceived discrimination and historical loss were positively and significantly associated with depressive symptoms and anxiety, whereas only perceived discrimination was positively and significantly associated with anger (all small to moderate effect sizes). |
| Whitbeck, et al., (2001). | Participants recruited from 3 reserves in the Upper Midwest of US. All 3 reservations had very minor regional variations, shared a common language, common spiritual beliefs, and common traditional practice. Each of the participating tribes provided a list of all enrolled families with a child in the eligible age range  Inclusion criteria: be enrolled in 5^th^-8^th^ grade (10-14 years). All families living on or within 50 miles of the reservations were invited to participate in the study. At least one parent and the selected target child had to participate to be counted.  N = 220 children (120 boys and 100 girls) who participated in a baseline survey for a prevention study.  Final samples was N = 195 participants (M*age* of the boys =12.2 years; the average age for girls =12.1 years | Examined whether the relation between discrimination and substance use in early adolescence was mediated by youths’ internalizing symptoms, externalizing symptoms, or anger. | Quantitative | Self-report, cross sectional survey.  Structural equation modelling was used to investigate potential mediators of the relationship between discrimination and substance abuse | Perceived discrimination was assessed as a latent construct made up of three factors derived from factor analysis of a ten-item dis- crimination scale. Response categories for the ten-item scale ranged from 1 = never to 3 = always. Global discrimination was a five item measure consisting of general experiences such as being ignored because of ethnicity, being excluded from activities, verbal insults, threats of harm and hearing racial slurs. Authority discrimination was made up of three items that included being treated disrespectfully by a store clerk, k, hassled by police because of ethnicity, or having adults suspect them of doing something wrong because they were American Indian. School discrimination consisted of two items concerning whether the adolescents' teachers had acted surprised when they did well or expected them not to do well because of their ethnicity. | Study designed in partnership with communities, Tribal Advisory Boards | Externalizing psychopathology (substance abuse; delinquent behaviour) Internalizing symptoms; anger. | Even though the respondents were in 5th through 8th grades, they had already experienced significant discrimination. Almost one- half (49%) the adolescents reported that they had been insulted because of being an American Indian. About one-fourth (23%) had felt disrespected in a place of business due to ethnicity. 49 percent had heard a racial slur yelled at them. 14 percent had been threatened physically. More than one-half reported that their teachers seemed surprised when they did well in school (54%); 25 percent felt their teachers did not expect them to do well on their schoolwork. One-fifth, (20%) felt the other children excluded them due to ethnicity and 31 percent felt that the other children treated them unfairly due to their being American Indian.  Girls and boys responded very similarly to the discrimination items (only statistically significant gender difference was that girls were more likely than boys to report that they had been disrespected in a place of business.)  There was a strong positive relationship between discrimination and substance abuse (I = .37, p < .01).  Perceived not discrimination was positively associated with internalization symptoms among the 5th through 8th grade adolescents (I = .36, p < .01) and with early substance abuse (I = .35, p < .01). When accounting for the effects of perceived discrimination, internalizing symptoms did not lead to substance abuse among this age group.  Internalizing did not mediate the relationship between early onset substance abuse and discrimination  erceived discrimination was strongly, positively related to feelings of anger among the adolescents (I = .41, p < .01). In turn, angry feelings were positively related to early onset substance abuse (1 = .21,p < .01). anger did not mediate the relationship between discrimination and sub- stance abuse  Feelings of anger and delinquent behavior in the model, the direct effect of PD though still statistically significant, was substantially reduced from a = .37 in the baseline model to a = .15 (p < .05), a reduction of 59 % abuse became nonsignificant. Anger was positively related to delinquent behavior (13 = .43, p < .01), and, in turn, delinquent behavior was strongly, positively related to early onset substance abuse (I = .47, p < .01). Discrimination was positively related to feelings of anger among the adolescents (13 = .43, p < .01) and to delinquent behavior (13 = .18, p < .05). As age of the adolescents increased they were more likely to report delinquent behavior (13 = .23,p < .01) and substance abuse (13 = .25,p < .01). The model explained 22 percent of the variance of adolescents' feelings of anger, 26 percent of the variance of delinquent behaviour, and 30 percent of the variance of early onset substance abuse. |
| Whitbeck et al., (2002). | N=189 Indigenous adolescents (115 boys; 97 girls). M*age* = 12.2 and 12.1 respectively. Same recruitment as Whitbeck. et al., (2001) | To test the hypothesis that gang involvement (GI) is positively related to a sense of hopelessness and alienation among minority adolescents.  Sub- Hypothesis that GI is related to PD. | Quantitative | Same as Whitbeck et al., (2001) | Same as Whitbeck et al., (2001) | Study designed in partnership with communities, Tribal Advisory Boards | Externalizing symptoms (gang involvement); hopelessness. | PD positively correlated with age of adolescent (r = .21), and mother anti-social history (r = .26), and hopelessness (r = .33), negatively correlated with family income (r = -.18), academic success (r = -.19), and attitudes toward school (r = -.29),  Participation in Traditional Activities slightly increased risk for gang involvement.  PD increased risk of gang involvement by 1.7 times (B = .51), controlling for several other covariates. |
| Zinga et al., (2016). | Recruited from a school who has a mixed cohort, with specialised programs and resources for Aboriginal students, funded by external bodies.  N = 44 students from a Secondary school in Canada (23 Caucasian; 14 female/ 7 male; and 21 Aboriginal 18 female/5 male).  All year levels. | Explore Aboriginal and Caucasian student perceptions of school experiences within a multicultural context.  Focus on juxtaposition between minority and majority students. | Qualitative | Mixed focus groups, ranging from 3 to 9 participants. Participants divided by level (applied or academic).  2 x all Aboriginal focus groups  5 x Majority Caucasian, with at least 1 Aboriginal student per group.  Open-ended focus group questions focused on students’ educational experiences, knowledge of multiculturalism and how it applied to education, knowledge of issues facing minority youth, and knowledge of the CRC.  Thematic analysis in NVivo – open thematic coding from repetitions in the data. | Acknowledges that racism is implicit and explicitly references racism in thematic results. | N/A | No explicit developmental outcomes delineated, but discussion concludes that:  “Racial discrimination, marginalisation, and cultural conflict are all affecting the positive developmental trajectories of students and young people”.  Additionally, the analysis of Aboriginal students refers to  Resiliency in coping strategies. | Racial discrimination, marginalisation, and cultural conflict are all affecting the positive developmental trajectories of students and young people in the context.  [racist] acts are very implicit and there is a common understanding that talking about race in any way is racism.  Coping responses to racism included Acceptance of lived reality and downplaying racist experiences., Repressing their race to fit in the white majority contexts they must navigate daily.  Authors conclude that “comments made by Aboriginal youth points to an ability to be resilient and work towards academic success even when facing implicit and explicit racism.”  Five themes emerged from thematic analysis: i) :Our school backs down from rules ; ii) Lack of awareness of Aboriginal issues (Caucasian dominant theme); iii) Reluctance to recognise Aboriginal culture (Caucasian dominant theme), iv) Racism is just in the community and very invested in downplaying it in their school (Both groups). V) If you talk about race, you are considered racist (Caucasian dominant theme). |

Notes: PD = perceived discrimination. SRE = Schedule of Racist Events. AOR = adjusted odds ratio.

**Supplementary Table 2 References**

Bodkin-Andrews, G. H., Craven, R. G. W., & Martin, A. J. (2006, July). Perceived discrimination and Indigenous Australian students’ school

related attitudes. In 4th International Biennial SELF Research Conference, Michigan.

Centers for Disease Control and Prevention. (2010). Youth risk behavior surveillance—United States, 2009. MMWR, 59, 1–142

King, M., Dinos, S., Shaw, J., Watson, R., Stevens, S., Passetti, F., ... & Serfaty, M. (2007). The Stigma Scale: development of a standardised

measure of the stigma of mental illness. *The British Journal of Psychiatry, 190*(3), 248-254.

Landrine, H., & Klonoff, E. A. (1996). The schedule of racist events: A measure of racial discrimination and a study of its negative physical and

mental health consequences. *Journal of Black Psychology*, *22*(2), 144-168.

Walters, K. (2009). Lessons from the field: race, culture and children’s mental health. Center for Excellence in Children’s Mental Health.

Wong, C. A., Eccles, J. S., & Sameroff, A. (2003). The influence of ethnic discrimination and ethnic identification on African American

adolescents' school and socioemotional adjustment. *Journal of personality, 71*(6), 1197-1232.

Yoder, K. (2001). Suicidal ideation among American Indian youth. *Dissertation Abstracts International: Section A, Humanities and Social*

*Sciences,* 62, 2247.
